# CT-Based Radiomics Enhance Respiratory Function Analysis for Lung SBRT

**DOI:** 10.3390/bioengineering12080800

**Published:** 2025-07-25

**Authors:** Alice Porazzi, Mattia Zaffaroni, Vanessa Eleonora Pierini, Maria Giulia Vincini, Aurora Gaeta, Sara Raimondi, Lucrezia Berton, Lars Johannes Isaksson, Federico Mastroleo, Sara Gandini, Monica Casiraghi, Gaia Piperno, Lorenzo Spaggiari, Juliana Guarize, Stefano Maria Donghi, Łukasz Kuncman, Roberto Orecchia, Stefania Volpe, Barbara Alicja Jereczek-Fossa

**Affiliations:** 1 Division of Radiation Oncology, IEO European Institute of Oncology IRCCS, 20141 Milan, Italymattia.zaffaroni@ircc.it (M.Z.); federico.mastroleo@ieo.it (F.M.); gaia.piperno@ieo.it (G.P.); stefania.volpe@ieo.it (S.V.); barbara.jereczek@ieo.it (B.A.J.-F.); 2 Department of Oncology and Hemato-Oncology, University of Milan, 20122 Milan, Italy; monica.casiraghi@ieo.it (M.C.); lorenzo.spaggiari@ieo.it (L.S.); 3 Department of Experimental Oncology, IEO European Institute of Experimental Oncology IRCCS, 20141 Milan, Italy; aurora.gaeta@ieo.it (A.G.); sara.gandini@ieo.it (S.G.); 4 Department of Statistics and Quantitative Methods, University of Milan-Bicocca, 20126 Milan, Italy; 5 Graduate School of Biomedical Omics, University of Milan, 20122 Milan, Italy; 6 Division of Thoracic Surgery, IEO European Institute of Oncology IRCCS, 20141 Milan, Italy; 7 Interventional Pneumology Unit, IEO European Institute of Oncology IRCCS, 20141 Milan, Italy; juliana.guarize@ieo.it (J.G.); stefanomaria.donghi@ieo.it (S.M.D.); 8 Department of Radiotherapy, Medical University of Lodz, 90-419 Lodz, Poland; lukasz.kuncman@umed.lodz.pl; 9 Department of External Beam Radiotherapy, Nicolaus Copernicus Multidisciplinary Centre for Oncology and Traumatology, 93-513 Lodz, Poland; 10 Scientific Directorate, IEO European Institute of Oncology IRCCS, 20141 Milan, Italy; roberto.orecchia@ieo.it

**Keywords:** radiomics, NSCLC, DL_CO_, biomarkers

## Abstract

**Introduction:** Radiomics is the extraction of non-invasive and reproducible quantitative imaging features, which may yield mineable information for clinical practice implementation. Quantification of lung function through radiomics could play a role in the management of patients with pulmonary lesions. The aim of this study is to test the capability of radiomic features to predict pulmonary function parameters, focusing on the diffusing capacity of lungs to carbon monoxide (DL_CO_). **Methods:** Retrospective data were retrieved from electronical medical records of patients treated with Stereotactic Body Radiation Therapy (SBRT) at a single institution. Inclusion criteria were as follows: (1) SBRT treatment performed for primary early-stage non-small cell lung cancer (ES-NSCLC) or oligometastatic lung nodules, (2) availability of simulation four-dimensional computed tomography (4DCT) scan, (3) baseline spirometry data availability, (4) availability of baseline clinical data, and (5) written informed consent for the anonymized use of data. The gross tumor volume (GTV) was segmented on 4DCT reconstructed phases representing the moment of maximum inhalation and maximum exhalation (Phase 0 and Phase 50, respectively), and radiomic features were extracted from the lung parenchyma subtracting the lesion/s. An iterative algorithm was clustered based on correlation, while keeping only those most associated with baseline and post-treatment DL_CO_. Three models were built to predict DL_CO_ abnormality: the clinical model—containing clinical information; the radiomic model—containing the radiomic score; the clinical-radiomic model—containing clinical information and the radiomic score. For the models just described, the following were constructed: Model 1 based on the features in Phase 0; Model 2 based on the features in Phase 50; Model 3 based on the difference between the two phases. The AUC was used to compare their performances. **Results:** A total of 98 patients met the inclusion criteria. The Charlson Comorbidity Index (CCI) scored as the clinical variable most associated with baseline DL_CO_ (*p* = 0.014), while the most associated features were mainly texture features and similar among the two phases. Clinical-radiomic models were the best at predicting both baseline and post-treatment abnormal DL_CO_. In particular, the performances for the three clinical-radiomic models at predicting baseline abnormal DL_CO_ were AUC_1_ = 0.72, AUC_2_ = 0.72, and AUC_3_ = 0.75, for Model 1, Model 2, and Model 3, respectively. Regarding the prediction of post-treatment abnormal DL_CO_, the performances of the three clinical-radiomic models were AUC_1_ = 0.91, AUC_2_ = 0.91, and AUC_3_ = 0.95, for Model 1, Model 2, and Model 3, respectively. **Conclusions:** This study demonstrates that radiomic features extracted from healthy lung parenchyma on a 4DCT scan are associated with baseline pulmonary function parameters, showing that radiomics can add a layer of information in surrogate models for lung function assessment. Preliminary results suggest the potential applicability of these models for predicting post-SBRT lung function, warranting validation in larger, prospective cohorts.

## 1. Introduction

In recent years, stereotactic body radiotherapy (SBRT) has emerged as a safe and effective treatment modality for a wide variety of clinical indications. On the one hand, it may be indicated as the primary treatment as an alternative to surgery, or when surgery is contraindicated due to underlying clinical conditions [1]. On the other hand, insights into the oligometastatic state have opened doors to metastasis-directed therapy (MDT), alone or in association with systemic treatments [2]. Specifically, results from phase II and III trials such as the SABR-COMET and the ORIOLE trials [3,4,5], have contributed to support the role of local consolidative therapies in patients with oligometastatic disease and controlled primary tumor, showing improvements in both overall survival and progression-free survival as compared to the standard of care, across multiple disease settings [6,7,8]. Arguably, such results are expected to be confirmed by other ongoing studies, while pre-clinical and translational research is focusing on the search for blood- and imaging-derived biomarkers for prognostic stratification and treatment response prediction [9].

In the thoracic region, the most frequent indications of SBRT currently include the treatment of primary lung lesions and lung oligometastases originating from either the lung or other primary sites (e.g., head and neck cancers, gynecological malignancies). Due to the current lack of evidence from randomized trials, the SBRT for primary lesions is mostly prescribed when surgery is deemed unfeasible, or when concerns about the potential functional consequences of resection exist [1]. However, available data support the use of SBRT in early-stage non-small cell lung cancer (ES-NSCLC) patients, showing excellent toxicity profiles and rates of local control [10,11,12]. The same excellent control rates have been shown for the treatment of oligometastatic lesions of the lungs, for a wide variety of primaries, including head and neck, lung, colorectal, breast cancers, and sarcomas [13,14,15].

While lung SBRT is safe and well tolerated even in highly comorbid patients, it is however advisable to estimate the pre-treatment pulmonary function, especially in those presenting with respiratory diseases, such as chronic obstructive pulmonary disease (COPD), or interstitial lung diseases, such as idiopathic lung fibrosis, or drug-induced interstitial lung disease [16,17]. Preliminary studies suggest that early changes in spirometry parameters, potentially predictive biomarkers for radiation pneumonitis, may warrant referral in cases of decreased lung function due to radiation-induced lung fibrosis [18]. However, albeit non-invasive, performing an adequate spirometry assessment requires sufficient patient compliance, and the exclusion of potential concomitant confounders, such as recent trauma, surgery, or infections [19].

Therefore, other non-invasive and indirect approaches are desirable in this context. Of these, radiomics is an appealing option [20]. It consists of the automated high-throughput extraction of large amounts of features from standard-of-care medical images. The hypothesis is that the quantitative analysis of macroscopic features on medical images can provide complementary information to those obtained by standard qualitative imaging analysis [21]. Features can be combined with non-imaging-based features and clinical data to build models that may predict some important patients’ parameters [22].

The aim of this study was to investigate the association between radiomic features extracted from the healthy lung parenchyma of each patient throughout four-dimensional computed tomography (4DCT) reconstructed phases representing the moment of maximum expiration and maximum inspiration, and baseline pulmonary function parameters, particularly focusing on the diffusing capacity to carbon monoxide (DL_CO_). Additionally, this association was assessed using post-SBRT spirometry parameters for a subset of patients for whom post-treatment spirometry data were available.

## 2. Patients and Methods

### 2.1. Clinical Dataset

This retrospective study considered patients treated between 2013 and 2022 in the RT Division at the European Institute of Oncology (IEO), IRCCS, Milan, Italy, based on the following inclusion criteria: (1) SBRT treatment performed at IEO, Milan, Italy for primary ES-NSCLC or oligometastatic lung nodules, (2) availability of simulation 4DCT scan, (3) baseline spirometry and clinical data availability, (4) written informed consent for the anonymized use of data for research purposes. The project was conducted in accordance with the principles outlined in the Declaration of Helsinki and has been approved and notified by the IEO Ethics Committee (code N93/11).

### 2.2. Imaging Dataset and Segmentation

For each patient, the analysis was performed on their simulation CT. All simulation CT scans were acquired with the same machine, a GE (General Electric Company, Boston, MA, USA) Medical System Optima CT 580 scanner. The scans were acquired in head-first supine position, helical mode with a slice thickness of 2.5 mm, tube voltage of 120 kV, and tube current set to 200 mA. The CT images were acquired with the 4DCT technique named “Gate 100”. The 4DCT simulation allows for the collection of CT scans over the course of respiration and for sorting them into bins based on either breathing amplitude or phase. The result is a series of images (typically 8–10 CTs) that reflect the target position at various points within the breathing cycle [23]. Automatic exposure control was not applied. Additional acquisition parameters included a revolution time of 1 s and a pitch of 0.652 to ensure sufficient oversampling of respiratory motion. Images were reconstructed through filtered back projection. Adaptive statistical iterative reconstruction (ASiR) was not performed. In this case, respiratory phase reconstruction was performed using the Advantage 4D software. In particular, Gate 100 enables the subdivision of the respiration cycle into nine phases, resulting in nine CT series.

The segmentations of the geometric GTV (gross tumor volume, i.e., visible/demonstrable extent and location of the disease) and entire lungs were both performed with the RayStation software (RaySearch Laboratories, Stockholm, Sweden, version 9B and version 11) by a radiation oncologist. The geometric GTV segmentation was performed manually by a single radiation oncologist. The GTV was geometrically expanded by 0.5 mm (GTV exp 0.50) to increase the reproducibility. The segmentation of the lungs was performed both on the contralateral and ipsilateral lungs with RayStation’s model-based segmentation (MBS) algorithm [24]. After the segmentation, the following volumes were taken from the CT scans representing the moment of maximum inhalation and maximum exhalation (in this case Phase 0 and Phase 50, respectively): both the lungs together (lungs), the lungs together subtracting the GTV (lungs-gtv), the ipsilateral lung subtracting the GTV (Lung_L/R-gtv). In cases of OMD patients with more than one metastatic lung lesion, this last volume was taken for each lesion present. Finally, the contours were saved in RT struct (RTSTRUCT) format.

### 2.3. Feature Extraction

The image preprocessing and feature extraction steps were performed using Python (version 3.7.16), with the following packages: PyRadiomics (version 3.0.1), NumPy (version 1.21.5), SimpleITK (version 2.1.1.2), and PyWavelet (version 1.3.0). Images (in DICOM files) were transformed into a SimpleITK data array in order to create structs and binary masks for all the patients using the coordinates of the contours of the ROIs. The masks obtained are used to read the structs using PyRadiomics. Before extracting radiomic features, the image intensities were normalized with histogram-based intensity normalization. Although all the images were acquired with the same scanner, histogram-based intensity normalization was performed to reduce variability due to differences in acquisition settings or patient-specific factors. A reference intensity distribution was defined using a CT scan as “standard”. Then, for each input CT scan, the intensity values corresponding to a set of predefined percentiles were computed and then linearly mapped to the corresponding values in the reference histogram. This process adjusts the image intensities such that the overall distribution aligns with the scan used as reference. The result, for each involved 4DCT scan in input, is an image with a rescaled intensity distribution that is more consistent across subjects and acquisition settings, enhancing the comparability and robustness of downstream radiomic features. For discretization, PyRadiomics’ default with absolute discretization with binWidth = 25 HU was used. After normalization and discretization, the feature extraction process was carried out using all PyRadiomics available feature classes (shape features, first-order features, and second-order features). Features were computed from both the original image and from images pre-processed with a range of built-in filters [25]. These include wavelet, Laplacian of Gaussian (LoG) filter, gradient, Local Binary Pattern (LBP)2D, LBP3D, square, square root, logarithm, and exponential filters. Wavelet and LoG filtering were implemented via the PyWavelets and SimpleITK libraries, respectively, while the remaining filters were applied using NumPy [25].

All extracted features were organized and returned in an ordered dictionary, which was also exported as a DataFrame in an Excel file. Each feature is uniquely identified by a name that includes the applied filter, feature class, and feature name.

### 2.4. Statistical Analysis

Frequencies, medians, and first and third quartiles were used to describe categorical and continuous variables, respectively. DL_CO_ was defined as normal if it was in the range of 75–140% [26]. Where appropriate, Wilcoxon’s test, Pearson’s chi-square test, and Fisher’s exact test were used to assess variables associated with abnormal DLco patients.

The univariate Wilcoxon signed-rank test was used to assess if, for those patients who had both baseline and post-treatment spirometry, the reduction in the pulmonary function parameters, observed between the baseline and post-treatment data was statistically significant.

Among the extracted features, features with near-zero variance and high correlation (Spearman > 0.95) were initially excluded. The remaining features were clustered by an iterative clustering algorithm, grouping features when the Spearman correlation coefficient was >0.75. In each cluster, only the feature mostly associated with abnormal DL_CO_ at baseline, and at post-treatment when available, was retained. The procedure was iterated until the correlation in finally selected features resulted in <0.75 and no more clusters were thus created. A coefficient for each feature was obtained by the logistic Least Absolute Shrinkage and Selection Operator (LASSO) Regression Model. Each coefficient represents the strength and direction of the association between a radiomic feature and the outcome. A positive coefficient indicates an increased likelihood of abnormal DL_CO_, while a negative coefficient suggests that higher values of the feature are associated with a decreased likelihood of abnormal DL_CO_. The resulting radiomic score for each patient was calculated by summing the feature value multiplied by its relative coefficient.

Clustering analysis and the LASSO logistic regression model were applied three times to develop three models: on the features extracted from Phase 0 (Phase 0), on the features extracted from Phase 50 (Phase 50), and finally on the difference of the features between Phase 0 and Phase 50 (∆ Phase).

### 2.5. Model Building

Three kinds of models were built to assess their capability at predicting DL_CO_ normal/abnormal classification at baseline and post-treatment: the clinical model—containing only clinical information; the radiomic model—containing only the radiomic score; clinical-radiomic model—containing both clinical information and a radiomic score. Odds ratio (OR) estimates were quantified, and 95% confidence intervals (CIs) were presented. In particular, three radiomic and three clinical-radiomic models were built to predict both baseline and post-treatment abnormal DL_CO_:

- the first radiomic model (Model 1) was built considering the radiomic features selected from Phase 0, the second one (Model 2) the radiomic features selected from Phase 50, and the third one (Model 3) the radiomic features selected from those that were different between the two phases;
- the three clinical-radiomic models were built following the same criteria, but considering the clinical variable selected as exemplary in each model.

**Figure 1:**
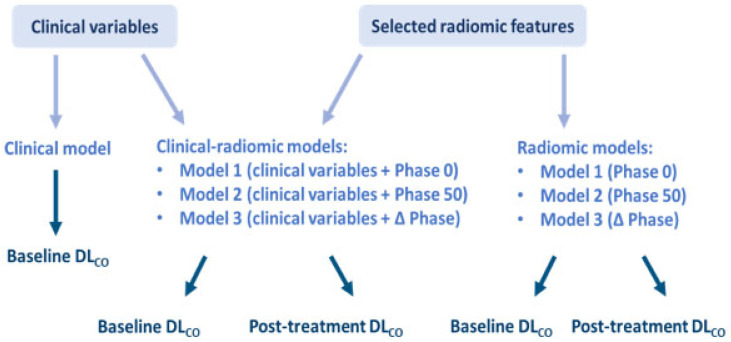
Schematic representation of the thirteen predictive models.

An additional clinical model, based on the single clinical variable selected, was built for predicting DL_CO_ classification only at the baseline DL_CO_, for a total of thirteen predictive models. A schematic representation of the developed predictive models is shown in Figure 1.

The performance of these models was evaluated with the method based on the Area Under the Curve (AUC). A repeated internal three-fold cross-validation was performed for each model. No external validation process was conducted. The statistical analyses were carried out using R version 4.3.1.

## 3. Results

### 3.1. Patient Characteristics

A total of 98 patients met the inclusion criteria. Of these patients, 43 were primary NSCLC cases, while the remaining 55 had been diagnosed with OMD from varying primary origins. The total number of nodules was 125, with a median lesion size (i.e., maximum diameter of the lesion) of 14 mm (IQR 10–19).

Patient-related characteristics and spirometric parameters are supplied in Supplementary Material—Table S1. Baseline spirometry was available for all 98 patients, while post-treatment spirometry was retrieved only for 24 patients. At baseline, a total of 43 patients had normal findings of DL_CO_, while 55 had abnormal values.

### 3.2. Associations

Associations between clinical variables and DL_CO_ values (normal vs. abnormal) are reported in Table 1. Among the main results, the Charlson Comorbidity Index (CCI, *p*-value = 0.014) was significantly higher in the abnormal DL_CO_ group. CCI was chosen arbitrarily among the associated variables as the most exemplary clinical variable to be used for model building. Other interesting statistically significant clinical variables correlated with abnormal baseline DL_CO_ are to be mentioned: COPD (*p* = 0.003), several comorbidities (*p* = 0.01), smoking habits (*p* = 0.05), and the origin of the tumor (primary or metastatic, *p* = <0.001).

**Figure 2:**
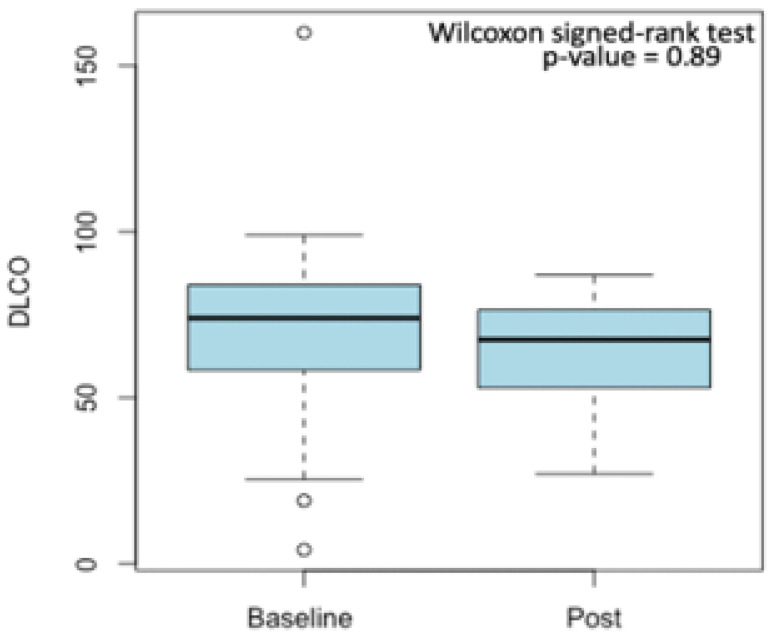
Reduction between median baseline and post-treatment DL_CO_ values. Outliers are displayed as small circles.

Median spirometry values were generally lower in post-treatment spirometry rather than in baseline spirometry data. Furthermore, for both basal and post-treatment spirometry, the median value of DL_CO_ was under the normality range. However, the univariate Wilcoxon signed-rank test, performed only considering the baseline and post-treatment pulmonary function values of the 24 patients for which both parameters were accessible, revealed that although a reduction between the median value of baseline and post-treatment DL_CO_ was observed (Figure 2), it was not statistically significant (*p* = 0.89).

### 3.3. Selected Radiomic Features

A total of 1982 radiomic features were extracted for each scan (Supplementary Material—Table S2). The same features were selected and included in the radiomic score calculation (Table 2), for Phase 0 and Phase 50 and their coefficients were very similar. In particular, the features selected for both Phase 0 and Phase 50 were second-order texture features obtained after applying two different matrices: the gray level co-occurrence matrix (GLCM) and the gray level dependence matrix (GLDM). Among the features selected for ∆ Phase, there were two features extracted applying the GLCM, three were first-order and a second-order feature was obtained upon the application of the gray level size zone matrix (GLSZM).

### 3.4. Model Performances

#### Baseline DL_CO_

**Figure 3:**
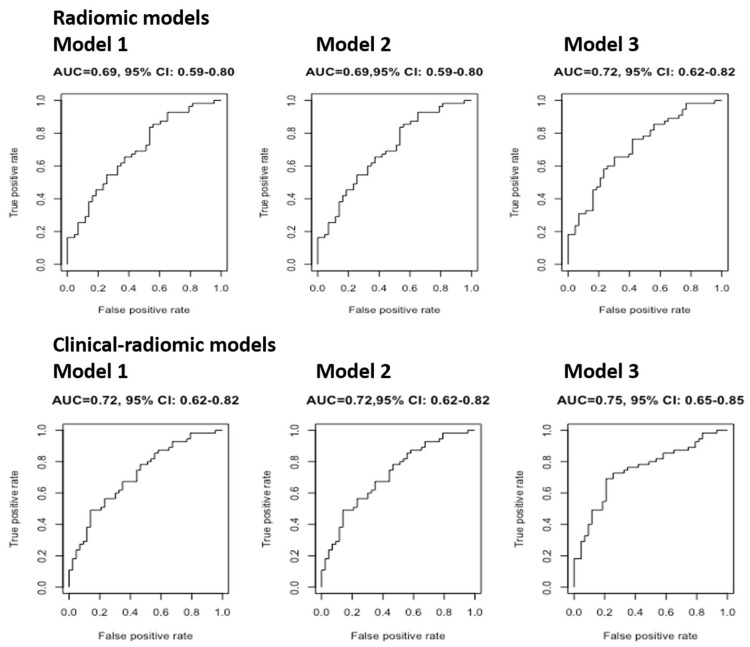
The AUC of radiomic and clinical-radiomic models (without cross-validation) for the prediction of baseline abnormal DLco.

The AUC of radiomic and clinical-radiomic models, before and after cross-validation, for the prediction of baseline abnormal DLco is displayed in Figure 3 and Table 3, respectively.

The median values of repeated cross-validation AUCs of the three radiomic models were all similar (0.69, 0,69, and 0.72, respectively), in concordance with the fact that even the coefficients of the radiomic features used were very similar. Model 3 explained 1% more than the other two models; however, since the AUC was not much higher than the AUC of the first two models, it could not be concluded that Model 3 is better than Models 1 and 2 at predicting abnormal DL_CO_ findings at baseline. Comparably, the three clinical-radiomic models showed similar AUC (0.71, 0.71, 0.74, respectively). In particular, it can be noted that these three models performed better than the respective three radiomic models, suggesting that CCI allowed for the attainment of a more performant predictive model. The clinical model showed the lowest AUC (0.65).

### 3.5. Post-Treatment DL_CO_

The AUC of radiomic and clinical-radiomic models, before and after cross-validation, for the prediction of post-treatment abnormal DLco is displayed in Supplementary Materials—Figure S1 and Table S3, respectively.

The median values of repeated cross-validation AUC for the three radiomic models and the three clinical-radiomic models were all similar (0.94, 0.94, 0.67, and 0.88, 0.88, 0.67, respectively). Both the radiomic and the clinical-radiomic model have a lower performance compared with the baseline setting. It has to be noted that the shape of the curves and the high values of AUC obtained were a consequence of the fact that post-treatment spirometry was available for only a minority of 24 patients, among which 10 and 14 had normal and abnormal values of DL_CO_, respectively.

## 4. Discussion

This study explored the potentiality of radiomics for building predictive models that could assess patients’ lung functionality and normal tissue response to SBRT. It investigated the association between spirometry parameters and radiomic features extracted from the lung parenchyma throughout 4DCT reconstructed phases corresponding to the moment of maximum inhalation and maximum exhalation. In particular, this project aimed at exploring the possibility of considering radiomic features as a non-invasive, quantitative, medical-image biomarker and at comparing the performance of radiomic models with those of clinical models and clinical-radiomic models at predicting baseline and post-treatment abnormal DL_CO_.

This means that the identification of stable features and clinical variables strongly associated with DL_CO_ is a fundamental step for the development of a radiomic signature able to describe the baseline and post-treatment lung functionality in clinics.

In particular, CCI was identified as the clinical variable most associated with the DL_CO_ status and included in the combined model (clinical-radiomic). It makes sense because CCI is evaluated considering a series of comorbidities. Considering a single clinical variable is usually not optimal, as it could lead to the building of a less-performing model. However, in this case, CCI was chosen as it is an index that summarizes different clinical conditions and pathologies. In fact, COPD and several comorbidities, which are among the parameters used to define CCI, were found to be statistically significantly correlated with abnormal DL_CO_ at univariate analysis. Among the other clinical variables previously identified as significant in the univariate analysis, the smoking habit was explained by the consideration that tobacco smoking reduces respiratory functions and was supported by the fact that the majority of smoking patients had an abnormal DL_CO_ (20 abnormal vs. 5 normal). Finally, regarding the origin of the tumor, it was observed that in patients with a primary origin, the number of these patients with an abnormal DL_CO_ was higher than the normal one. This may suggest that primary lung cancers are more prone to worsen the lung function than metastatic lung cancers, as in the latter situation lungs are not initially affected by the disease, but in a second moment of cancer evolution.

On the other hand, the cluster analysis and LASSO selected the features most associated with abnormal DL_CO_ findings for three phases: Phase 0, Phase 50, and ∆ Phase. The features selected for Phase 0 and Phase 50 and the calculated coefficients were very similar. Furthermore, the features resulting from this analysis were mainly second-order texture features, advising that a texture analysis of lung images is associated with lung functionality, and it can be used to characterize the complexity of lung tissue and pathological processes. In our study, most of the radiomic features retained after the selection process were second-order texture features, particularly derived from the Gray Level Co-occurrence Matrix (GLCM) and the Gray Level Dependence Matrix (GLDM). These features quantify heterogeneity, spatial relationships, and textural complexity within the healthy lung parenchyma. While a definitive biological interpretation remains challenging, one possible explanation for their association with DLco is that DLco reflects the efficiency of gas exchange across the alveolar-capillary membrane and may be influenced by subclinical alterations in lung microstructure. Such changes—related to alveolar integrity, interstitial remodeling, or vascular alterations—might subtly affect the CT image texture. Features such as GLCM Correlation and GLDM Low Gray Level Emphasis could therefore be sensitive to these subtle tissue characteristics, potentially contributing to the differentiation between normal and abnormal DLco. However, further biological validation is needed to confirm these hypotheses. In addition, it can be observed that all but two of the LASSO-selected radiomic features are derived from filtered images. This is consistent with the fact that using filters is a strategy to enhance image properties and to unveil otherwise undetectable information. This suggests that the use of image filtering can be considered among the strengths of this study, also given that it has been previously shown that radiomic features derived from preprocessed CTs are associated with oncological outcomes in cancers from other districts [27,28].

Finally, the AUC was evaluated to assess the performance of the radiomic, clinical-radiomic, and clinical predictive models built for both abnormal baseline and post-treatment DL_CO_.

The clinical-radiomic models proved to be better than the clinical model at predicting abnormal baseline DL_CO_, suggesting that radiomics can provide an additional layer of information. This advised that, although CCI is a good clinical surrogate, as it summarizes many pathological conditions, a predictive model built on a single clinical variable is less performing. However clinical-radiomic models were the best-performing ones and in particular Model 3, suggesting that the variation of the features extracted from Phase 0 and Phase 50 adds a small, yet appreciable boost, with respect to Model 1 and Model 2.

Even among the models predicting abnormal post-treatment DL_CO_, the clinical-radiomic models performed best, although in this case Model 3 was the worst. This might be explained considering that models predicting abnormal post-treatment DL_CO_ were based on data retrieved by only 24 patients, suggesting that these results are to be evaluated with caution and require further investigations and a larger cohort of patients. However, the fact that the radiomic features that differ among Phases 0 and 50 are highly informative at predicting an abnormal baseline DL_CO_ and at the same time, less informative at predicting an abnormal post-treatment DL_CO_, requires additional examinations.

In addition, it can be speculated that the higher AUC values of post-treatment DL_CO_ might be due to a model overfitting, given the high number of variables for a small cohort of 24 patients for which post-treatment spirometry data were available.

To conclude, this study supported the importance of integrating radiomic features into clinical variables in the building of models, which can be useful in predicting lung functionality. In concordance with this finding, comparable results were reported by Sawayanagi et al., who proposed clinical-radiomic prediction models in the setting of ES-NSCLC patients treated with SBRT [29]. As in this series, radiomic features were extracted from simulation CTs acquired at free-breathing using PyRadiomics, although the authors did not enable all filtering modalities but focused solely on the permutations of the wavelet. In particular, they found an association between overall survival and one high-order feature, which allowed for an efficient prognostic stratification in the validation cohort, supporting that texture seems to predict patients’ outcomes, at least in terms of survival.

To the best of our knowledge, some studies have investigated the association between pulmonary functions and radiomic features in the context of pulmonary disorders [30,31,32], but only Lafta et al. have analyzed the relation between pulmonary functions and lung cancer [33]. For example, Zhou et al. recently published a retrospective study performed on a larger cohort of 2785 patients in which they investigated the role of CT-based whole lung radiomics in differentiating COPD patients from non-COPD patients [34]. Specifically, patients who underwent pulmonary function testing in five hospitals were divided into a non-COPD group and a COPD group. The radiomic features of the whole lung volume were extracted and 18 of them were selected by LASSO logistic regression to construct a radiomic model. Then, similar to our study, they compared the performance of three types of models: the radiomic model, the clinical model, and the nomogram constructed by combining the radiomic score, age, sex, height, and smoking status. In agreement with our results, they found that the latter had the best performance and accuracy in identifying COPD [34]. Finally, an additional advantage of this study is represented by the high quality of the image dataset, which presents homogenous acquisition parameters and segmentations performed by a single observer.

The present study is not exempt from relevant limitations. Firstly, a primary source of uncertainty lies in the small size of the dataset. Although the study spans a substantial period (2013–2022), only a limited number of patients met the inclusion criteria. This aspect constrained the statistical power and limited the generalizability of our findings. Consequently, the results of the present study should be interpreted with caution and warrant further validation in larger, more comprehensive datasets. We are aware that the insights derived are insufficient to draw definitive or broadly generalizable conclusions. This is even more relevant considering post-treatment DLco, as post-treatment spirometry is not explicitly recommended by current clinical practice guidelines.

Additionally, the absence of an independent validation cohort further restricts the confidence in the findings. Our study relied solely on internal validation through repeated cross-validation within a single-institution cohort. While this method provides an estimate of model performance and stability within the development dataset, it does not directly assess generalizability to external populations or different imaging protocols. Future perspectives include performing external validation using an independent dataset from a different institution or time period to evaluate the robustness and generalizability of our findings. Future steps also include performing a SHapley Additive exPlanations (SHAP) analysis [35] to further enhance the evaluation and interpretability of the models. This analysis enables the quantification of the contribution of each input feature to the model’s predictions. By leveraging this approach—widely adopted in recent studies [36,37,38]—we aim to gain deeper insights into the influence of specific features, thereby improving the transparency and clinical relevance of the predictive models.

Furthermore, the study specifically focused on predicting DLco, as it reflects the integrity of the alveolar-capillary interface and the efficiency of gas exchange—physiological processes more likely to be associated with textural alterations detectable by radiomic analysis. In contrast, spirometric parameters primarily reflect airway mechanics and volumetric capacities, which are less likely to be directly captured by CT-based radiomic features. Given the exploratory nature of the study and the absence of external validation, we deliberately limited the analysis to a single functional endpoint that we considered physiologically coherent with CT-derived features. This approach aimed to reduce the risk of spurious associations and improve interpretability. However, we acknowledge that including additional parameters, such as FEV or FVC, could have added further value to the present analysis.

Another limitation is related to the segmentation process. GTVs were contoured by a single experienced radiation oncologist, to ensure consistency and avoid inter-observer variability, which can significantly affect radiomic feature extraction and model performance. However, we acknowledge that this approach may limit the generalizability of the findings and does not account for person-dependent variability in clinical practice. Moreover, the lung segmentation was performed with a model-based algorithm, whose performance required manual intervention, especially in the lower portion of the volume due to the presence of the diaphragm. To strengthen the reliability of future analyses, a more advanced and fully automated segmentation approach should be considered. Lastly, the small lesion size could have affected the reliability of certain radiomic features, and although class imbalance was minimal (43 vs. 55 cases), it was not explicitly addressed during model development.

## 5. Conclusions and Future Perspectives

This study demonstrates that radiomic features extracted from healthy lung parenchyma on a 4DCT scan are associated with baseline pulmonary function parameters, showing that radiomics can add a layer of information in surrogate models for lung function assessment. Although post-treatment spirometry data were limited, preliminary results suggest the potential applicability of these models for predicting post-SBRT lung function, warranting validation in larger, prospective cohorts.

Radiomics could offer a promising, non-invasive tool for pulmonary function prediction in SBRT candidates, with potential to support personalized treatment planning and risk stratification, particularly when conventional pulmonary function testing is not feasible or inconclusive. The future perspective of this project concerns expanding the analysis on a larger, more complex, and comprehensive cohort.

## Figures and Tables

**Table 1:** Associations between clinical variables and DL_CO_ values (normal vs. abnormal). Significant *p*-values are highlighted in bold.

	Characteristics	*p*-Value ^1^
Baseline	Sex	>0.9
	Age at diagnosis	0.2
	Comorbidity	**0.01**
	CCI	**0.014**
	Arterial hypertension	**0.046**
	Cardiopathy	0.075
	Diabetes	0.4
	COPD	**0.003**
	Smoking habit	**0.05**
	Hb	0.4
	CRP	0.9
	Oxygen Therapy	>0.9
	SpO2 basal	**<0.001**
	PRIM/M+	**<0.001**
	M+ origin	0.11
	Histology	**0.01**
	Histological type	**>0.9**
	Number of lesions	0.3
	Side of the lesions	0.6
	Central/peripheral lesion	0.2
	Shape	0.13
	Margin	0.7
	Diam max (mm)	0.2
	Systemic concomitant therapy	0.7
	Type of systemic therapy	>0.9
	RE-RT	0.7
	Number of fractions	**0.004**
	Dose/fraction	**0.008**
	Total prescribed dose	0.1
Baseline spirometry data	VC	0.2
	FEV_1_	**0.009**
	PEF	0.057
	VC%	**<0.001**
	FEV_1_%	**<0.001**
Post-treatment spirometry data	VC	>0.9
	FEV_1_	0.2
	PEF	0.9
	VC%	**0.035**
	FEV_1_%	**<0.001**
Follow-up	Status	0.2

^1^ Wilcoxon rank sum test; Pearson’s Chi-squared test; Fisher’s exact test; Wilcoxon rank sum exact test.

**Table 2:** Radiomic features selected and included in the radiomic score models and their coefficients.

	Radiomic Features	Coefficient
Phase 0	wavelet-HHH GLCM Correlation	−39.20
	wavelet-LLL GLDM Low Gray Level Emphasis	57.85
Phase 50	wavelet-HHH GLCM Correlation	−38.56
	wavelet-LLL GLDM Low Gray Level Emphasis	54.49
∆ Phase	Gradient GLSZM Size Zone Non Uniformity Normalized	0.346
	log-sigma-2-0-mm-3D First Order Maximum	0.00023
	wavelet-HLL First Order Maximum	2.35
	wavelet-HHL First Order Median	0.14
	wavelet-HHH GLCM Correlation	1.21
	wavelet-HHH GLCM Imc1	−2.61

**Table 3:** Median value of repeated cross-validation AUC for models predicting abnormal baseline DLco.

		Repeated Cross-Validation of AUC (Median, IQR)
Radiomic Models	Model 1	0.69 (0.67–0.71)
	Model 2	0.69 (0.67–0.71)
	Model 3	0.72 (0.71–0.74)
Clinical- Radiomic Models	Model 1	0.71 (0.69–0.73)
	Model 2	0.71 (0.69–0.73)
	Model 3	0.74 (0.71–0.76)
Clinical Model		0.65 (0.63–0.67)

## Data Availability

The data presented in this study are not publicly available and are available on request from the corresponding author.

## Supplementary Materials

The following supporting information can be downloaded at: https://www.mdpi.com/article/10.3390/bioengineering12080800/s1, Table S1: Cohort clinical data, characteristics, and spirometry data. Table S2: Extracted radiomic features. Table S3: Median value of repeated cross-validation AUC for models predicting abnormal baseline and post-treatment DL_CO_. Figure S1: AUC of radiomic and clinical-radiomic models (without cross-validation) for the prediction of post-treatment abnormal DLco.

## Abbreviations

4DCT	Four-Dimensional Computed Tomography
AUC	Area Under the Curve
CCI	Charlson Comorbidity Index
CI	Confidence Interval
COPD	Chronic Obstructive Pulmonary Disease
DL_CO_	Diffusing Capacity to Carbon Monoxide
ECIS	European Cancer Information System
ESMO	European Society for Medical Oncology
ES	Early stage
FEV_1_	Forced Expiratory Volume 1
GLCM	Gray Leve Co-Occurrence Matrix
GLDM	Gray Level Dependence Matrix
GLSZM	Gray Level Size Zone Matrix
FVC	Forced Vital Capacity
GTV	Gross Tumor Volume
LASSO	Least Absolute Shrinkage and Selection Operator
NSCLC	Non-Small Cell Lung Cancer
OMD	Oligometastatic Disease
PFTs	Pulmonary Function Tests
PMD	Polymetastatic Disease
ROI	Region of Interest
SBRT	Stereotactic Body Radiotherapy
VOI	Volume of Interest
