# Supplementary material for: CT-Based Radiomics Enhance Respiratory Function Analysis for Lung SBRT
Source: Bioengineering (Basel). 2025 Jul 25;12(8):800. doi: 10.3390/bioengineering12080800 (PMC12384004; doi:10.3390/bioengineering12080800)
Supplement: Supplementary file 1 [file bioengineering-12-00800-s001.zip › bioengineering-3680644-supplementary.pdf]

Supplementary material Table S1 - Cohort clinical data, characteristics and spirometry data.

	Characteristics		Overall, N = 98[1]	Normal, N = 43[1]	Abnormal, N = 55[1]
			n (%)	n (%)	n (%)
Baseline	Sex				
		men	57 (58%)	25 (58%)	32 (58%)
		women	41 (42%)	18 (42%)	23 (42%)
	Comorbidity		68 (69%)	24 (56%)	44 (80%)
	Hypertension		43 (44%)	14 (33%)	29 (53%)
	Cardiopathy		22 (22%)	6 (14%)	16 (29%)
	Diabetes		12 (12%)	4 (9.3%)	8 (15%)
	COPD		14 (14%)	1 (2.3%)	13 (24%)
	Smoking habit				
		no	28 (29%)	14 (45%)	14 (30%)
		yes	25 (25%)	5 (16%)	20 (43%)
		previous	25 (25%)	12 (39%)	13 (28%)
		missing	20 (21%)	12	8
	PRIM/M+				
		PRIM	43 (44%)	10 (23%)	33 (60%)
		M+	55 (56%)	33 (77%)	22 (40%)
	M+ origin				
		Urological	11 (20%)	7 (21%)	4 (18%)
		Breast	4 (7.3%)	3 (9.1%)	1 (4.5%)
		Gastro-intestinal	20 (36%)	12 (36%)	8 (36%)
		Ginecological	11 (20%)	3 (9.1%)	8 (36%)
		Head/Neck	5 (9.1%)	5 (15%)	0 (0%)
		Sarcoma/Memalnoma	4 (7.3%)	3 (9.1%)	1 (4.5%)
		Unknown	43	10	33
	Systemic concomitant therapy		7 (7.1%)	4 (9.3%)	3 (5.5%)
	Type of systemic therapy				
		Hormonal Therapy	3 (43%)	2 (50%)	1 (33%)
		Monoclonal Antibody	1 (14%)	0 (0%)	1 (33%)
		Monoclonal Antibody+Chemotherapy	1 (14%)	1 (25%)	0 (0%)
		Chemotherapy	1 (14%)	0 (0%)	1 (33%)
		PARP inhibitor	1 (14%)	1 (25%)	0 (0%)
		Unknown	91	39	52
	RE-RT				
		no	96 (98%)	42 (98%)	54 (98%)
		yes	2 (2.0%)	1 (2.3%)	1 (1.8%)
	CRP				
		0.1	3 (30%)	1 (50%)	2 (25%)
		0.2	1 (10%)	0 (0%)	1 (12%)
		0.8	1 (10%)	0 (0%)	1 (12%)
		1.9	2 (20%)	0 (0%)	2 (25%)
		2.5	1 (10%)	0 (0%)	1 (12%)
		2.6	1 (10%)	0 (0%)	1 (12%)
		3.94	1 (10%)	1 (50%)	0 (0%)
		Unknown	88	41	47
	Oxygen Therapy		3 (4.3%)	1 (3.4%)	2 (4.9%)

	Unknown	28	14	14
	Number of lesions			
	1	76 (78%)	30 (70%)	46 (84%)
	2	18 (18%)	10 (23%)	8 (15%)
	3	3 (3.1%)	2 (4.7%)	1 (1.8%)
	4	1 (1.0%)	1 (2.3%)	0 (0%)
	Side of the lesions			
	Right	50 (51%)	21 (49%)	29 (53%)
	Right+Left	8 (8.2%)	5 (12%)	3 (5.5%)
	Left	40 (41%)	17 (40%)	23 (42%)
	Central/peripheral lesion			
	Central	26 (27%)	12 (28%)	14 (26%)
	Central+Peripheral	10 (10%)	7 (16%)	3 (5.6%)
	Peripheral	61 (63%)	24 (56%)	37 (69%)
	Unknown	1	0	1
	Shape			
	Round	23 (51%)	10 (67%)	13 (43%)
	Complex	13 (29%)	2 (13%)	11 (37%)
	Oval	8 (18%)	2 (13%)	6 (20%)
	Complex/Oval	1 (2.2%)	1 (6.7%)	0 (0%)
	Unknown	53	28	25
	Margin			
	Smooth	7 (25%)	2 (40%)	5 (22%)
	Lobulated	2 (7.1%)	0 (0%)	2 (8.7%)
	Irregular	19 (68%)	3 (60%)	16 (70%)
	Unknown	70	38	32
	Histology	21 (21%)	4 (9.3%)	17 (31%)
	Histological type			
	Adenocarcinomas	13 (62%)	3 (75%)	10 (59%)
	Squamous	5 (24%)	1 (25%)	4 (24%)
	NOS (no otherwise specified)	3 (14%)	0 (0%)	3 (18%)
	Unknown	77	39	38
	Number of fractions			
	3	67 (68%)	23 (53%)	44 (80%)
	4	1 (1.0%)	0 (0%)	1 (1.8%)
	5	30 (31%)	20 (47%)	10 (18%)
		Median (IQR)	Median (IQR)	Median (IQR)
	Age at diagnosis	70 (61, 79)	67 (58, 78)	72 (66, 79)
	CCI	6.00 (5.00, 7.00)	6.00 (4.00, 6.00)	6.00 (5.00, 7.00)
	Hb	13.20 (12.60, 14.30)	13.00 (12.60, 14.25)	13.40 (12.88, 15.22)
	Unknown	55	24	31
	SpO_2 basal	97.00 (96.00, 98.00)	98.00 (97.00, 98.00)	97.00 (95.00, 97.00)
	Unknown	33	14	19
	Diam max (mm)	14 (10, 19)	12 (9, 19)	15 (12, 19)
	Unknown	3	1	2
	Dose/fraction	15.0 (10.0, 18.0)	15.0 (9.5, 15.0)	15.0 (13.5, 18.0)
	Total prescribed dose	45 (45, 54)	45 (40, 50)	48 (45, 54)
Baseline spirometry data		n (%)	n (%)	n (%)
	Basal spirometry availability	98 (100%)	43 (44%)	55 (54%)
		Median (IQR)	Median (IQR)	Median (IQR)

	VC	2.95 (2.08, 3.41)	3.11 (2.09, 3.69)	2.74 (2.00, 3.36)
	Unknown	30	20	10
	FEV_1	2.04 (1.44, 2.47)	2.30 (1.82, 2.72)	1.89 (1.23, 2.36)
	Unknown	12	7	5
	PEF	64 (45, 83)	76 (64, 92)	57 (44, 78)
	Unknown	31	21	10
	DL_{CO}	74 (59, 84)	85 (78, 88)	60 (47, 72)
	VC%	90 (77, 103)	101 (92, 108)	84 (73, 95)
	Unknown	32	21	11
	FEV_1%	89 (68, 102)	100 (90, 108)	80 (60, 96)
	Unknown	10	7	3
	DL_{CO}/VA	89 (68, 102)	100 (93, 115)	81 (61, 95)
	Unknown	24	17	7
Post-treatment spirometry data		n (%)	n (%)	n (%)
	Post-treatment spirometry availability	24 (24.5%)	10 (10.5%)	14 (14%)
		Median (IQR)	Median (IQR)	Median (IQR)
	VC	2.14 (1.86, 2.75)	1.90 (1.84, 3.09)	2.19 (1.90, 2.52)
	Unknown	76	36	40
	FEV_1	1.65 (1.20, 2.07)	2.03 (1.48, 2.52)	1.61 (0.94, 2.04)
	Unknown	76	36	40
	PEF	63 (41, 84)	69 (46, 81)	58 (44, 82)
	Unknown	78	37	41
	DL_{CO}	68 (54, 76)	76 (74, 80)	59 (47, 74)
	Unknown	74	35	39
	VC%	84 (72, 89)	92 (87, 101)	76 (68, 88)
	Unknown	78	37	41
	FEV_1%	72 (54, 87)	90 (89, 97)	64 (46, 73)
	Unknown	78	37	41
	DL_{CO}/VA	83 (76, 100)	100 (87, 108)	81 (74, 95)
	Unknown	79	37	42
Follow up		n (%)	n (%)	n (%)
	Status			
	NED	48 (49%)	19 (44%)	29 (53%)
	AWD	37 (38%)	20 (47%)	17 (31%)
	DEAD	13 (13%)	4 (9.3%)	9 (16%)

[1] Median (IQR); Range; n (%)

Supplementary material Table S2 – Extracted radiomic features

diagnostics_Image-original_Hash
diagnostics_Image-original_Dimensionality
diagnostics_Image-original_Spacing
diagnostics_Image-original_Size
diagnostics_Image-original_Mean
diagnostics_Image-original_Minimum
diagnostics_Image-original_Maximum
diagnostics_Mask-original_Hash
diagnostics_Mask-original_Spacing
diagnostics_Mask-original_Size
diagnostics_Mask-original_BoundingBox
diagnostics_Mask-original_VoxelNum
diagnostics_Mask-original_VolumeNum
diagnostics_Mask-original_CenterOfMassIndex
diagnostics_Mask-original_CenterOfMass
original_shape_Elongation
original_shape_Flatness
original_shape_LeastAxisLength
original_shape_MajorAxisLength
original_shape_Maximum2DDiameterColumn
original_shape_Maximum2DDiameterRow
original_shape_Maximum2DDiameterSlice
original_shape_Maximum3DDiameter
original_shape_MeshVolume
original_shape_MinorAxisLength
original_shape_Sphericity
original_shape_SurfaceArea
original_shape_SurfaceVolumeRatio
original_shape_VoxelVolume
original_firstorder_10Percentile
original_firstorder_90Percentile
original_firstorder_Energy
original_firstorder_Entropy
original_firstorder_InterquartileRange
original_firstorder_Kurtosis
original_firstorder_Maximum
original_firstorder_MeanAbsoluteDeviation
original_firstorder_Mean
original_firstorder_Median
original_firstorder_Minimum
original_firstorder_Range
original_firstorder_RobustMeanAbsoluteDeviation
original_firstorder_RootMeanSquared
original_firstorder_Skewness
original_firstorder_TotalEnergy
original_firstorder_Uniformity
original_firstorder_Variance
original_glcm_Autocorrelation
original_glcm_ClusterProminence

original_glcm_ClusterShade
original_glcm_ClusterTendency
original_glcm_Contrast
original_glcm_Correlation
original_glcm_DifferenceAverage
original_glcm_DifferenceEntropy
original_glcm_DifferenceVariance
original_glcm_Id
original_glcm_Idm
original_glcm_Idmn
original_glcm_Idn
original_glcm_Imc1
original_glcm_Imc2
original_glcm_InverseVariance
original_glcm_JointAverage
original_glcm_JointEnergy
original_glcm_JointEntropy
original_glcm_MCC
original_glcm_MaximumProbability
original_glcm_SumAverage
original_glcm_SumEntropy
original_glcm_SumSquares
original_gldm_DependenceEntropy
original_gldm_DependenceNonUniformity
original_gldm_DependenceNonUniformityNormalized
original_gldm_DependenceVariance
original_gldm_GrayLevelNonUniformity
original_gldm_GrayLevelVariance
original_gldm_HighGrayLevelEmphasis
original_gldm_LargeDependenceEmphasis
original_gldm_LargeDependenceHighGrayLevelEmphasis
original_gldm_LargeDependenceLowGrayLevelEmphasis
original_gldm_LowGrayLevelEmphasis
original_gldm_SmallDependenceEmphasis
original_gldm_SmallDependenceHighGrayLevelEmphasis
original_gldm_SmallDependenceLowGrayLevelEmphasis
original_glrlm_GrayLevelNonUniformity
original_glrlm_GrayLevelNonUniformityNormalized
original_glrlm_GrayLevelVariance
original_glrlm_HighGrayLevelRunEmphasis
original_glrlm_LongRunEmphasis
original_glrlm_LongRunHighGrayLevelEmphasis
original_glrlm_LongRunLowGrayLevelEmphasis
original_glrlm_LowGrayLevelRunEmphasis
original_glrlm_RunEntropy
original_glrlm_RunLengthNonUniformity
original_glrlm_RunLengthNonUniformityNormalized
original_glrlm_RunPercentage
original_glrlm_RunVariance
original_glrlm_ShortRunEmphasis
original_glrlm_ShortRunHighGrayLevelEmphasis
original_glrlm_ShortRunLowGrayLevelEmphasis
original_glszm_GrayLevelNonUniformity

original_glszm_GrayLevelNonUniformityNormalized
original_glszm_GrayLevelVariance
original_glszm_HighGrayLevelZoneEmphasis
original_glszm_LargeAreaEmphasis
original_glszm_LargeAreaHighGrayLevelEmphasis
original_glszm_LargeAreaLowGrayLevelEmphasis
original_glszm_LowGrayLevelZoneEmphasis
original_glszm_SizeZoneNonUniformity
original_glszm_SizeZoneNonUniformityNormalized
original_glszm_SmallAreaEmphasis
original_glszm_SmallAreaHighGrayLevelEmphasis
original_glszm_SmallAreaLowGrayLevelEmphasis
original_glszm_ZoneEntropy
original_glszm_ZonePercentage
original_glszm_ZoneVariance
original_ngtdm_Busyness
original_ngtdm_Coarseness
original_ngtdm_Complexity
original_ngtdm_Contrast
original_ngtdm_Strength
exponential_firstorder_10Percentile
exponential_firstorder_90Percentile
exponential_firstorder_Energy
exponential_firstorder_Entropy
exponential_firstorder_InterquartileRange
exponential_firstorder_Kurtosis
exponential_firstorder_Maximum
exponential_firstorder_MeanAbsoluteDeviation
exponential_firstorder_Mean
exponential_firstorder_Median
exponential_firstorder_Minimum
exponential_firstorder_Range
exponential_firstorder_RobustMeanAbsoluteDeviation
exponential_firstorder_RootMeanSquared
exponential_firstorder_Skewness
exponential_firstorder_TotalEnergy
exponential_firstorder_Uniformity
exponential_firstorder_Variance
exponential_glcm_Autocorrelation
exponential_glcm_ClusterProminence
exponential_glcm_ClusterShade
exponential_glcm_ClusterTendency
exponential_glcm_Contrast
exponential_glcm_Correlation
exponential_glcm_DifferenceAverage
exponential_glcm_DifferenceEntropy
exponential_glcm_DifferenceVariance
exponential_glcm_Id
exponential_glcm_Idm
exponential_glcm_Idmn
exponential_glcm_Idn
exponential_glcm_Imc1
exponential_glcm_Imc2

exponential_glcm_InverseVariance
exponential_glcm_JointAverage
exponential_glcm_JointEnergy
exponential_glcm_JointEntropy
exponential_glcm_MCC
exponential_glcm_MaximumProbability
exponential_glcm_SumAverage
exponential_glcm_SumEntropy
exponential_glcm_SumSquares
exponential_gldm_DependenceEntropy
exponential_gldm_DependenceNonUniformity
exponential_gldm_DependenceNonUniformityNormalized
exponential_gldm_DependenceVariance
exponential_gldm_GrayLevelNonUniformity
exponential_gldm_GrayLevelVariance
exponential_gldm_HighGrayLevelEmphasis
exponential_gldm_LargeDependenceEmphasis
exponential_gldm_LargeDependenceHighGrayLevelEmphasis
exponential_gldm_LargeDependenceLowGrayLevelEmphasis
exponential_gldm_LowGrayLevelEmphasis
exponential_gldm_SmallDependenceEmphasis
exponential_gldm_SmallDependenceHighGrayLevelEmphasis
exponential_gldm_SmallDependenceLowGrayLevelEmphasis
exponential_glrlm_GrayLevelNonUniformity
exponential_glrlm_GrayLevelNonUniformityNormalized
exponential_glrlm_GrayLevelVariance
exponential_glrlm_HighGrayLevelRunEmphasis
exponential_glrlm_LongRunEmphasis
exponential_glrlm_LongRunHighGrayLevelEmphasis
exponential_glrlm_LongRunLowGrayLevelEmphasis
exponential_glrlm_LowGrayLevelRunEmphasis
exponential_glrlm_RunEntropy
exponential_glrlm_RunLengthNonUniformity
exponential_glrlm_RunLengthNonUniformityNormalized
exponential_glrlm_RunPercentage
exponential_glrlm_RunVariance
exponential_glrlm_ShortRunEmphasis
exponential_glrlm_ShortRunHighGrayLevelEmphasis
exponential_glrlm_ShortRunLowGrayLevelEmphasis
exponential_glszm_GrayLevelNonUniformity
exponential_glszm_GrayLevelNonUniformityNormalized
exponential_glszm_GrayLevelVariance
exponential_glszm_HighGrayLevelZoneEmphasis
exponential_glszm_LargeAreaEmphasis
exponential_glszm_LargeAreaHighGrayLevelEmphasis
exponential_glszm_LargeAreaLowGrayLevelEmphasis
exponential_glszm_LowGrayLevelZoneEmphasis
exponential_glszm_SizeZoneNonUniformity
exponential_glszm_SizeZoneNonUniformityNormalized
exponential_glszm_SmallAreaEmphasis
exponential_glszm_SmallAreaHighGrayLevelEmphasis
exponential_glszm_SmallAreaLowGrayLevelEmphasis
exponential_glszm_ZoneEntropy

exponential_glszm_ZonePercentage
exponential_glszm_ZoneVariance
exponential_ngtdm_Busyness
exponential_ngtdm_Coarseness
exponential_ngtdm_Complexity
exponential_ngtdm_Contrast
exponential_ngtdm_Strength
gradient_firstorder_10Percentile
gradient_firstorder_90Percentile
gradient_firstorder_Energy
gradient_firstorder_Entropy
gradient_firstorder_InterquartileRange
gradient_firstorder_Kurtosis
gradient_firstorder_Maximum
gradient_firstorder_MeanAbsoluteDeviation
gradient_firstorder_Mean
gradient_firstorder_Median
gradient_firstorder_Minimum
gradient_firstorder_Range
gradient_firstorder_RobustMeanAbsoluteDeviation
gradient_firstorder_RootMeanSquared
gradient_firstorder_Skewness
gradient_firstorder_TotalEnergy
gradient_firstorder_Uniformity
gradient_firstorder_Variance
gradient_glcm_Autocorrelation
gradient_glcm_ClusterProminence
gradient_glcm_ClusterShade
gradient_glcm_ClusterTendency
gradient_glcm_Contrast
gradient_glcm_Correlation
gradient_glcm_DifferenceAverage
gradient_glcm_DifferenceEntropy
gradient_glcm_DifferenceVariance
gradient_glcm_Id
gradient_glcm_Idm
gradient_glcm_Idmn
gradient_glcm_Idn
gradient_glcm_Imc1
gradient_glcm_Imc2
gradient_glcm_InverseVariance
gradient_glcm_JointAverage
gradient_glcm_JointEnergy
gradient_glcm_JointEntropy
gradient_glcm_MCC
gradient_glcm_MaximumProbability
gradient_glcm_SumAverage
gradient_glcm_SumEntropy
gradient_glcm_SumSquares
gradient_gldm_DependenceEntropy
gradient_gldm_DependenceNonUniformity
gradient_gldm_DependenceNonUniformityNormalized
gradient_gldm_DependenceVariance

gradient_gldm_GrayLevelNonUniformity
gradient_gldm_GrayLevelVariance
gradient_gldm_HighGrayLevelEmphasis
gradient_gldm_LargeDependenceEmphasis
gradient_gldm_LargeDependenceHighGrayLevelEmphasis
gradient_gldm_LargeDependenceLowGrayLevelEmphasis
gradient_gldm_LowGrayLevelEmphasis
gradient_gldm_SmallDependenceEmphasis
gradient_gldm_SmallDependenceHighGrayLevelEmphasis
gradient_gldm_SmallDependenceLowGrayLevelEmphasis
gradient_glrlm_GrayLevelNonUniformity
gradient_glrlm_GrayLevelNonUniformityNormalized
gradient_glrlm_GrayLevelVariance
gradient_glrlm_HighGrayLevelRunEmphasis
gradient_glrlm_LongRunEmphasis
gradient_glrlm_LongRunHighGrayLevelEmphasis
gradient_glrlm_LongRunLowGrayLevelEmphasis
gradient_glrlm_LowGrayLevelRunEmphasis
gradient_glrlm_RunEntropy
gradient_glrlm_RunLengthNonUniformity
gradient_glrlm_RunLengthNonUniformityNormalized
gradient_glrlm_RunPercentage
gradient_glrlm_RunVariance
gradient_glrlm_ShortRunEmphasis
gradient_glrlm_ShortRunHighGrayLevelEmphasis
gradient_glrlm_ShortRunLowGrayLevelEmphasis
gradient_glszm_GrayLevelNonUniformity
gradient_glszm_GrayLevelNonUniformityNormalized
gradient_glszm_GrayLevelVariance
gradient_glszm_HighGrayLevelZoneEmphasis
gradient_glszm_LargeAreaEmphasis
gradient_glszm_LargeAreaHighGrayLevelEmphasis
gradient_glszm_LargeAreaLowGrayLevelEmphasis
gradient_glszm_LowGrayLevelZoneEmphasis
gradient_glszm_SizeZoneNonUniformity
gradient_glszm_SizeZoneNonUniformityNormalized
gradient_glszm_SmallAreaEmphasis
gradient_glszm_SmallAreaHighGrayLevelEmphasis
gradient_glszm_SmallAreaLowGrayLevelEmphasis
gradient_glszm_ZoneEntropy
gradient_glszm_ZonePercentage
gradient_glszm_ZoneVariance
gradient_ngtdm_Busyness
gradient_ngtdm_Coarseness
gradient_ngtdm_Complexity
gradient_ngtdm_Contrast
gradient_ngtdm_Strength
lbp-2D_firstorder_10Percentile
lbp-2D_firstorder_90Percentile
lbp-2D_firstorder_Energy
lbp-2D_firstorder_Entropy
lbp-2D_firstorder_InterquartileRange
lbp-2D_firstorder_Kurtosis

lbp-2D_firstorder_Maximum
lbp-2D_firstorder_MeanAbsoluteDeviation
lbp-2D_firstorder_Mean
lbp-2D_firstorder_Median
lbp-2D_firstorder_Minimum
lbp-2D_firstorder_Range
lbp-2D_firstorder_RobustMeanAbsoluteDeviation
lbp-2D_firstorder_RootMeanSquared
lbp-2D_firstorder_Skewness
lbp-2D_firstorder_TotalEnergy
lbp-2D_firstorder_Uniformity
lbp-2D_firstorder_Variance
lbp-2D_glcm_Autocorrelation
lbp-2D_glcm_ClusterProminence
lbp-2D_glcm_ClusterShade
lbp-2D_glcm_ClusterTendency
lbp-2D_glcm_Contrast
lbp-2D_glcm_Correlation
lbp-2D_glcm_DifferenceAverage
lbp-2D_glcm_DifferenceEntropy
lbp-2D_glcm_DifferenceVariance
lbp-2D_glcm_Id
lbp-2D_glcm_Idm
lbp-2D_glcm_Idmn
lbp-2D_glcm_Idn
lbp-2D_glcm_Imc1
lbp-2D_glcm_Imc2
lbp-2D_glcm_InverseVariance
lbp-2D_glcm_JointAverage
lbp-2D_glcm_JointEnergy
lbp-2D_glcm_JointEntropy
lbp-2D_glcm_MCC
lbp-2D_glcm_MaximumProbability
lbp-2D_glcm_SumAverage
lbp-2D_glcm_SumEntropy
lbp-2D_glcm_SumSquares
lbp-2D_gldm_DependenceEntropy
lbp-2D_gldm_DependenceNonUniformity
lbp-2D_gldm_DependenceNonUniformityNormalized
lbp-2D_gldm_DependenceVariance
lbp-2D_gldm_GrayLevelNonUniformity
lbp-2D_gldm_GrayLevelVariance
lbp-2D_gldm_HighGrayLevelEmphasis
lbp-2D_gldm_LargeDependenceEmphasis
lbp-2D_gldm_LargeDependenceHighGrayLevelEmphasis
lbp-2D_gldm_LargeDependenceLowGrayLevelEmphasis
lbp-2D_gldm_LowGrayLevelEmphasis
lbp-2D_gldm_SmallDependenceEmphasis
lbp-2D_gldm_SmallDependenceHighGrayLevelEmphasis
lbp-2D_gldm_SmallDependenceLowGrayLevelEmphasis
lbp-2D_glrlm_GrayLevelNonUniformity
lbp-2D_glrlm_GrayLevelNonUniformityNormalized
lbp-2D_glrlm_GrayLevelVariance

lbp-2D_glrlm_HighGrayLevelRunEmphasis
lbp-2D_glrlm_LongRunEmphasis
lbp-2D_glrlm_LongRunHighGrayLevelEmphasis
lbp-2D_glrlm_LongRunLowGrayLevelEmphasis
lbp-2D_glrlm_LowGrayLevelRunEmphasis
lbp-2D_glrlm_RunEntropy
lbp-2D_glrlm_RunLengthNonUniformity
lbp-2D_glrlm_RunLengthNonUniformityNormalized
lbp-2D_glrlm_RunPercentage
lbp-2D_glrlm_RunVariance
lbp-2D_glrlm_ShortRunEmphasis
lbp-2D_glrlm_ShortRunHighGrayLevelEmphasis
lbp-2D_glrlm_ShortRunLowGrayLevelEmphasis
lbp-2D_glszm_GrayLevelNonUniformity
lbp-2D_glszm_GrayLevelNonUniformityNormalized
lbp-2D_glszm_GrayLevelVariance
lbp-2D_glszm_HighGrayLevelZoneEmphasis
lbp-2D_glszm_LargeAreaEmphasis
lbp-2D_glszm_LargeAreaHighGrayLevelEmphasis
lbp-2D_glszm_LargeAreaLowGrayLevelEmphasis
lbp-2D_glszm_LowGrayLevelZoneEmphasis
lbp-2D_glszm_SizeZoneNonUniformity
lbp-2D_glszm_SizeZoneNonUniformityNormalized
lbp-2D_glszm_SmallAreaEmphasis
lbp-2D_glszm_SmallAreaHighGrayLevelEmphasis
lbp-2D_glszm_SmallAreaLowGrayLevelEmphasis
lbp-2D_glszm_ZoneEntropy
lbp-2D_glszm_ZonePercentage
lbp-2D_glszm_ZoneVariance
lbp-2D_ngtdm_Busyness
lbp-2D_ngtdm_Coarseness
lbp-2D_ngtdm_Complexity
lbp-2D_ngtdm_Contrast
lbp-2D_ngtdm_Strength
lbp-3D-m1_firstorder_10Percentile
lbp-3D-m1_firstorder_90Percentile
lbp-3D-m1_firstorder_Energy
lbp-3D-m1_firstorder_Entropy
lbp-3D-m1_firstorder_InterquartileRange
lbp-3D-m1_firstorder_Kurtosis
lbp-3D-m1_firstorder_Maximum
lbp-3D-m1_firstorder_MeanAbsoluteDeviation
lbp-3D-m1_firstorder_Mean
lbp-3D-m1_firstorder_Median
lbp-3D-m1_firstorder_Minimum
lbp-3D-m1_firstorder_Range
lbp-3D-m1_firstorder_RobustMeanAbsoluteDeviation
lbp-3D-m1_firstorder_RootMeanSquared
lbp-3D-m1_firstorder_Skewness
lbp-3D-m1_firstorder_TotalEnergy
lbp-3D-m1_firstorder_Uniformity
lbp-3D-m1_firstorder_Variance
lbp-3D-m1_glcm_Autocorrelation

lbp-3D-m1_glcm_ClusterProminence
lbp-3D-m1_glcm_ClusterShade
lbp-3D-m1_glcm_ClusterTendency
lbp-3D-m1_glcm_Contrast
lbp-3D-m1_glcm_Correlation
lbp-3D-m1_glcm_DifferenceAverage
lbp-3D-m1_glcm_DifferenceEntropy
lbp-3D-m1_glcm_DifferenceVariance
lbp-3D-m1_glcm_Id
lbp-3D-m1_glcm_Idm
lbp-3D-m1_glcm_Idmn
lbp-3D-m1_glcm_Idn
lbp-3D-m1_glcm_Imc1
lbp-3D-m1_glcm_Imc2
lbp-3D-m1_glcm_InverseVariance
lbp-3D-m1_glcm_JointAverage
lbp-3D-m1_glcm_JointEnergy
lbp-3D-m1_glcm_JointEntropy
lbp-3D-m1_glcm_MCC
lbp-3D-m1_glcm_MaximumProbability
lbp-3D-m1_glcm_SumAverage
lbp-3D-m1_glcm_SumEntropy
lbp-3D-m1_glcm_SumSquares
lbp-3D-m1_gldm_DependenceEntropy
lbp-3D-m1_gldm_DependenceNonUniformity
lbp-3D-m1_gldm_DependenceNonUniformityNormalized
lbp-3D-m1_gldm_DependenceVariance
lbp-3D-m1_gldm_GrayLevelNonUniformity
lbp-3D-m1_gldm_GrayLevelVariance
lbp-3D-m1_gldm_HighGrayLevelEmphasis
lbp-3D-m1_gldm_LargeDependenceEmphasis
lbp-3D-m1_gldm_LargeDependenceHighGrayLevelEmphasis
lbp-3D-m1_gldm_LargeDependenceLowGrayLevelEmphasis
lbp-3D-m1_gldm_LowGrayLevelEmphasis
lbp-3D-m1_gldm_SmallDependenceEmphasis
lbp-3D-m1_gldm_SmallDependenceHighGrayLevelEmphasis
lbp-3D-m1_gldm_SmallDependenceLowGrayLevelEmphasis
lbp-3D-m1_glrlm_GrayLevelNonUniformity
lbp-3D-m1_glrlm_GrayLevelNonUniformityNormalized
lbp-3D-m1_glrlm_GrayLevelVariance
lbp-3D-m1_glrlm_HighGrayLevelRunEmphasis
lbp-3D-m1_glrlm_LongRunEmphasis
lbp-3D-m1_glrlm_LongRunHighGrayLevelEmphasis
lbp-3D-m1_glrlm_LongRunLowGrayLevelEmphasis
lbp-3D-m1_glrlm_LowGrayLevelRunEmphasis
lbp-3D-m1_glrlm_RunEntropy
lbp-3D-m1_glrlm_RunLengthNonUniformity
lbp-3D-m1_glrlm_RunLengthNonUniformityNormalized
lbp-3D-m1_glrlm_RunPercentage
lbp-3D-m1_glrlm_RunVariance
lbp-3D-m1_glrlm_ShortRunEmphasis
lbp-3D-m1_glrlm_ShortRunHighGrayLevelEmphasis
lbp-3D-m1_glrlm_ShortRunLowGrayLevelEmphasis

lbp-3D-m1_glszm_GrayLevelNonUniformity
lbp-3D-m1_glszm_GrayLevelNonUniformityNormalized
lbp-3D-m1_glszm_GrayLevelVariance
lbp-3D-m1_glszm_HighGrayLevelZoneEmphasis
lbp-3D-m1_glszm_LargeAreaEmphasis
lbp-3D-m1_glszm_LargeAreaHighGrayLevelEmphasis
lbp-3D-m1_glszm_LargeAreaLowGrayLevelEmphasis
lbp-3D-m1_glszm_LowGrayLevelZoneEmphasis
lbp-3D-m1_glszm_SizeZoneNonUniformity
lbp-3D-m1_glszm_SizeZoneNonUniformityNormalized
lbp-3D-m1_glszm_SmallAreaEmphasis
lbp-3D-m1_glszm_SmallAreaHighGrayLevelEmphasis
lbp-3D-m1_glszm_SmallAreaLowGrayLevelEmphasis
lbp-3D-m1_glszm_ZoneEntropy
lbp-3D-m1_glszm_ZonePercentage
lbp-3D-m1_glszm_ZoneVariance
lbp-3D-m1_ngtdm_Busyness
lbp-3D-m1_ngtdm_Coarseness
lbp-3D-m1_ngtdm_Complexity
lbp-3D-m1_ngtdm_Contrast
lbp-3D-m1_ngtdm_Strength
lbp-3D-m2_firstorder_10Percentile
lbp-3D-m2_firstorder_90Percentile
lbp-3D-m2_firstorder_Energy
lbp-3D-m2_firstorder_Entropy
lbp-3D-m2_firstorder_InterquartileRange
lbp-3D-m2_firstorder_Kurtosis
lbp-3D-m2_firstorder_Maximum
lbp-3D-m2_firstorder_MeanAbsoluteDeviation
lbp-3D-m2_firstorder_Mean
lbp-3D-m2_firstorder_Median
lbp-3D-m2_firstorder_Minimum
lbp-3D-m2_firstorder_Range
lbp-3D-m2_firstorder_RobustMeanAbsoluteDeviation
lbp-3D-m2_firstorder_RootMeanSquared
lbp-3D-m2_firstorder_Skewness
lbp-3D-m2_firstorder_TotalEnergy
lbp-3D-m2_firstorder_Uniformity
lbp-3D-m2_firstorder_Variance
lbp-3D-m2_glcm_Autocorrelation
lbp-3D-m2_glcm_ClusterProminence
lbp-3D-m2_glcm_ClusterShade
lbp-3D-m2_glcm_ClusterTendency
lbp-3D-m2_glcm_Contrast
lbp-3D-m2_glcm_Correlation
lbp-3D-m2_glcm_DifferenceAverage
lbp-3D-m2_glcm_DifferenceEntropy
lbp-3D-m2_glcm_DifferenceVariance
lbp-3D-m2_glcm_Id
lbp-3D-m2_glcm_Idm
lbp-3D-m2_glcm_Idmn
lbp-3D-m2_glcm_Idn
lbp-3D-m2_glcm_Imc1

lbp-3D-m2_glcm_Imc2
lbp-3D-m2_glcm_InverseVariance
lbp-3D-m2_glcm_JointAverage
lbp-3D-m2_glcm_JointEnergy
lbp-3D-m2_glcm_JointEntropy
lbp-3D-m2_glcm_MCC
lbp-3D-m2_glcm_MaximumProbability
lbp-3D-m2_glcm_SumAverage
lbp-3D-m2_glcm_SumEntropy
lbp-3D-m2_glcm_SumSquares
lbp-3D-m2_gldm_DependenceEntropy
lbp-3D-m2_gldm_DependenceNonUniformity
lbp-3D-m2_gldm_DependenceNonUniformityNormalized
lbp-3D-m2_gldm_DependenceVariance
lbp-3D-m2_gldm_GrayLevelNonUniformity
lbp-3D-m2_gldm_GrayLevelVariance
lbp-3D-m2_gldm_HighGrayLevelEmphasis
lbp-3D-m2_gldm_LargeDependenceEmphasis
lbp-3D-m2_gldm_LargeDependenceHighGrayLevelEmphasis
lbp-3D-m2_gldm_LargeDependenceLowGrayLevelEmphasis
lbp-3D-m2_gldm_LowGrayLevelEmphasis
lbp-3D-m2_gldm_SmallDependenceEmphasis
lbp-3D-m2_gldm_SmallDependenceHighGrayLevelEmphasis
lbp-3D-m2_gldm_SmallDependenceLowGrayLevelEmphasis
lbp-3D-m2_glrlm_GrayLevelNonUniformity
lbp-3D-m2_glrlm_GrayLevelNonUniformityNormalized
lbp-3D-m2_glrlm_GrayLevelVariance
lbp-3D-m2_glrlm_HighGrayLevelRunEmphasis
lbp-3D-m2_glrlm_LongRunEmphasis
lbp-3D-m2_glrlm_LongRunHighGrayLevelEmphasis
lbp-3D-m2_glrlm_LongRunLowGrayLevelEmphasis
lbp-3D-m2_glrlm_LowGrayLevelRunEmphasis
lbp-3D-m2_glrlm_RunEntropy
lbp-3D-m2_glrlm_RunLengthNonUniformity
lbp-3D-m2_glrlm_RunLengthNonUniformityNormalized
lbp-3D-m2_glrlm_RunPercentage
lbp-3D-m2_glrlm_RunVariance
lbp-3D-m2_glrlm_ShortRunEmphasis
lbp-3D-m2_glrlm_ShortRunHighGrayLevelEmphasis
lbp-3D-m2_glrlm_ShortRunLowGrayLevelEmphasis
lbp-3D-m2_glszm_GrayLevelNonUniformity
lbp-3D-m2_glszm_GrayLevelNonUniformityNormalized
lbp-3D-m2_glszm_GrayLevelVariance
lbp-3D-m2_glszm_HighGrayLevelZoneEmphasis
lbp-3D-m2_glszm_LargeAreaEmphasis
lbp-3D-m2_glszm_LargeAreaHighGrayLevelEmphasis
lbp-3D-m2_glszm_LargeAreaLowGrayLevelEmphasis
lbp-3D-m2_glszm_LowGrayLevelZoneEmphasis
lbp-3D-m2_glszm_SizeZoneNonUniformity
lbp-3D-m2_glszm_SizeZoneNonUniformityNormalized
lbp-3D-m2_glszm_SmallAreaEmphasis
lbp-3D-m2_glszm_SmallAreaHighGrayLevelEmphasis
lbp-3D-m2_glszm_SmallAreaLowGrayLevelEmphasis

lbp-3D-m2_glszm_ZoneEntropy
lbp-3D-m2_glszm_ZonePercentage
lbp-3D-m2_glszm_ZoneVariance
lbp-3D-m2_ngtdm_Busyness
lbp-3D-m2_ngtdm_Coarseness
lbp-3D-m2_ngtdm_Complexity
lbp-3D-m2_ngtdm_Contrast
lbp-3D-m2_ngtdm_Strength
lbp-3D-k_firstorder_10Percentile
lbp-3D-k_firstorder_90Percentile
lbp-3D-k_firstorder_Energy
lbp-3D-k_firstorder_Entropy
lbp-3D-k_firstorder_InterquartileRange
lbp-3D-k_firstorder_Kurtosis
lbp-3D-k_firstorder_Maximum
lbp-3D-k_firstorder_MeanAbsoluteDeviation
lbp-3D-k_firstorder_Mean
lbp-3D-k_firstorder_Median
lbp-3D-k_firstorder_Minimum
lbp-3D-k_firstorder_Range
lbp-3D-k_firstorder_RobustMeanAbsoluteDeviation
lbp-3D-k_firstorder_RootMeanSquared
lbp-3D-k_firstorder_Skewness
lbp-3D-k_firstorder_TotalEnergy
lbp-3D-k_firstorder_Uniformity
lbp-3D-k_firstorder_Variance
lbp-3D-k_glcm_Autocorrelation
lbp-3D-k_glcm_ClusterProminence
lbp-3D-k_glcm_ClusterShade
lbp-3D-k_glcm_ClusterTendency
lbp-3D-k_glcm_Contrast
lbp-3D-k_glcm_Correlation
lbp-3D-k_glcm_DifferenceAverage
lbp-3D-k_glcm_DifferenceEntropy
lbp-3D-k_glcm_DifferenceVariance
lbp-3D-k_glcm_Id
lbp-3D-k_glcm_Idm
lbp-3D-k_glcm_Idmn
lbp-3D-k_glcm_Idn
lbp-3D-k_glcm_Imc1
lbp-3D-k_glcm_Imc2
lbp-3D-k_glcm_InverseVariance
lbp-3D-k_glcm_JointAverage
lbp-3D-k_glcm_JointEnergy
lbp-3D-k_glcm_JointEntropy
lbp-3D-k_glcm_MCC
lbp-3D-k_glcm_MaximumProbability
lbp-3D-k_glcm_SumAverage
lbp-3D-k_glcm_SumEntropy
lbp-3D-k_glcm_SumSquares
lbp-3D-k_gldm_DependenceEntropy
lbp-3D-k_gldm_DependenceNonUniformity
lbp-3D-k_gldm_DependenceNonUniformityNormalized

lbp-3D-k_gldm_DependenceVariance
lbp-3D-k_gldm_GrayLevelNonUniformity
lbp-3D-k_gldm_GrayLevelVariance
lbp-3D-k_gldm_HighGrayLevelEmphasis
lbp-3D-k_gldm_LargeDependenceEmphasis
lbp-3D-k_gldm_LargeDependenceHighGrayLevelEmphasis
lbp-3D-k_gldm_LargeDependenceLowGrayLevelEmphasis
lbp-3D-k_gldm_LowGrayLevelEmphasis
lbp-3D-k_gldm_SmallDependenceEmphasis
lbp-3D-k_gldm_SmallDependenceHighGrayLevelEmphasis
lbp-3D-k_gldm_SmallDependenceLowGrayLevelEmphasis
lbp-3D-k_glrlm_GrayLevelNonUniformity
lbp-3D-k_glrlm_GrayLevelNonUniformityNormalized
lbp-3D-k_glrlm_GrayLevelVariance
lbp-3D-k_glrlm_HighGrayLevelRunEmphasis
lbp-3D-k_glrlm_LongRunEmphasis
lbp-3D-k_glrlm_LongRunHighGrayLevelEmphasis
lbp-3D-k_glrlm_LongRunLowGrayLevelEmphasis
lbp-3D-k_glrlm_LowGrayLevelRunEmphasis
lbp-3D-k_glrlm_RunEntropy
lbp-3D-k_glrlm_RunLengthNonUniformity
lbp-3D-k_glrlm_RunLengthNonUniformityNormalized
lbp-3D-k_glrlm_RunPercentage
lbp-3D-k_glrlm_RunVariance
lbp-3D-k_glrlm_ShortRunEmphasis
lbp-3D-k_glrlm_ShortRunHighGrayLevelEmphasis
lbp-3D-k_glrlm_ShortRunLowGrayLevelEmphasis
lbp-3D-k_glszm_GrayLevelNonUniformity
lbp-3D-k_glszm_GrayLevelNonUniformityNormalized
lbp-3D-k_glszm_GrayLevelVariance
lbp-3D-k_glszm_HighGrayLevelZoneEmphasis
lbp-3D-k_glszm_LargeAreaEmphasis
lbp-3D-k_glszm_LargeAreaHighGrayLevelEmphasis
lbp-3D-k_glszm_LargeAreaLowGrayLevelEmphasis
lbp-3D-k_glszm_LowGrayLevelZoneEmphasis
lbp-3D-k_glszm_SizeZoneNonUniformity
lbp-3D-k_glszm_SizeZoneNonUniformityNormalized
lbp-3D-k_glszm_SmallAreaEmphasis
lbp-3D-k_glszm_SmallAreaHighGrayLevelEmphasis
lbp-3D-k_glszm_SmallAreaLowGrayLevelEmphasis
lbp-3D-k_glszm_ZoneEntropy
lbp-3D-k_glszm_ZonePercentage
lbp-3D-k_glszm_ZoneVariance
lbp-3D-k_ngtdm_Busyness
lbp-3D-k_ngtdm_Coarseness
lbp-3D-k_ngtdm_Complexity
lbp-3D-k_ngtdm_Contrast
lbp-3D-k_ngtdm_Strength
log-sigma-1-0-mm-3D_firstorder_10Percentile
log-sigma-1-0-mm-3D_firstorder_90Percentile
log-sigma-1-0-mm-3D_firstorder_Energy
log-sigma-1-0-mm-3D_firstorder_Entropy
log-sigma-1-0-mm-3D_firstorder_InterquartileRange

log-sigma-1-0-mm-3D_firstorder_Kurtosis
log-sigma-1-0-mm-3D_firstorder_Maximum
log-sigma-1-0-mm-3D_firstorder_MeanAbsoluteDeviation
log-sigma-1-0-mm-3D_firstorder_Mean
log-sigma-1-0-mm-3D_firstorder_Median
log-sigma-1-0-mm-3D_firstorder_Minimum
log-sigma-1-0-mm-3D_firstorder_Range
log-sigma-1-0-mm-3D_firstorder_RobustMeanAbsoluteDeviation
log-sigma-1-0-mm-3D_firstorder_RootMeanSquared
log-sigma-1-0-mm-3D_firstorder_Skewness
log-sigma-1-0-mm-3D_firstorder_TotalEnergy
log-sigma-1-0-mm-3D_firstorder_Uniformity
log-sigma-1-0-mm-3D_firstorder_Variance
log-sigma-1-0-mm-3D_glcm_Autocorrelation
log-sigma-1-0-mm-3D_glcm_ClusterProminence
log-sigma-1-0-mm-3D_glcm_ClusterShade
log-sigma-1-0-mm-3D_glcm_ClusterTendency
log-sigma-1-0-mm-3D_glcm_Contrast
log-sigma-1-0-mm-3D_glcm_Correlation
log-sigma-1-0-mm-3D_glcm_DifferenceAverage
log-sigma-1-0-mm-3D_glcm_DifferenceEntropy
log-sigma-1-0-mm-3D_glcm_DifferenceVariance
log-sigma-1-0-mm-3D_glcm_Id
log-sigma-1-0-mm-3D_glcm_Idm
log-sigma-1-0-mm-3D_glcm_Idmn
log-sigma-1-0-mm-3D_glcm_Idn
log-sigma-1-0-mm-3D_glcm_Imc1
log-sigma-1-0-mm-3D_glcm_Imc2
log-sigma-1-0-mm-3D_glcm_InverseVariance
log-sigma-1-0-mm-3D_glcm_JointAverage
log-sigma-1-0-mm-3D_glcm_JointEnergy
log-sigma-1-0-mm-3D_glcm_JointEntropy
log-sigma-1-0-mm-3D_glcm_MCC
log-sigma-1-0-mm-3D_glcm_MaximumProbability
log-sigma-1-0-mm-3D_glcm_SumAverage
log-sigma-1-0-mm-3D_glcm_SumEntropy
log-sigma-1-0-mm-3D_glcm_SumSquares
log-sigma-1-0-mm-3D_gldm_DependenceEntropy
log-sigma-1-0-mm-3D_gldm_DependenceNonUniformity
log-sigma-1-0-mm-3D_gldm_DependenceNonUniformityNormalized
log-sigma-1-0-mm-3D_gldm_DependenceVariance
log-sigma-1-0-mm-3D_gldm_GrayLevelNonUniformity
log-sigma-1-0-mm-3D_gldm_GrayLevelVariance
log-sigma-1-0-mm-3D_gldm_HighGrayLevelEmphasis
log-sigma-1-0-mm-3D_gldm_LargeDependenceEmphasis
log-sigma-1-0-mm-3D_gldm_LargeDependenceHighGrayLevelEmphasis
log-sigma-1-0-mm-3D_gldm_LargeDependenceLowGrayLevelEmphasis
log-sigma-1-0-mm-3D_gldm_LowGrayLevelEmphasis
log-sigma-1-0-mm-3D_gldm_SmallDependenceEmphasis
log-sigma-1-0-mm-3D_gldm_SmallDependenceHighGrayLevelEmphasis
log-sigma-1-0-mm-3D_gldm_SmallDependenceLowGrayLevelEmphasis
log-sigma-1-0-mm-3D_glrlm_GrayLevelNonUniformity
log-sigma-1-0-mm-3D_glrlm_GrayLevelNonUniformityNormalized

log-sigma-1-0-mm-3D_glrlm_GrayLevelVariance
log-sigma-1-0-mm-3D_glrlm_HighGrayLevelRunEmphasis
log-sigma-1-0-mm-3D_glrlm_LongRunEmphasis
log-sigma-1-0-mm-3D_glrlm_LongRunHighGrayLevelEmphasis
log-sigma-1-0-mm-3D_glrlm_LongRunLowGrayLevelEmphasis
log-sigma-1-0-mm-3D_glrlm_LowGrayLevelRunEmphasis
log-sigma-1-0-mm-3D_glrlm_RunEntropy
log-sigma-1-0-mm-3D_glrlm_RunLengthNonUniformity
log-sigma-1-0-mm-3D_glrlm_RunLengthNonUniformityNormalized
log-sigma-1-0-mm-3D_glrlm_RunPercentage
log-sigma-1-0-mm-3D_glrlm_RunVariance
log-sigma-1-0-mm-3D_glrlm_ShortRunEmphasis
log-sigma-1-0-mm-3D_glrlm_ShortRunHighGrayLevelEmphasis
log-sigma-1-0-mm-3D_glrlm_ShortRunLowGrayLevelEmphasis
log-sigma-1-0-mm-3D_glszm_GrayLevelNonUniformity
log-sigma-1-0-mm-3D_glszm_GrayLevelNonUniformityNormalized
log-sigma-1-0-mm-3D_glszm_GrayLevelVariance
log-sigma-1-0-mm-3D_glszm_HighGrayLevelZoneEmphasis
log-sigma-1-0-mm-3D_glszm_LargeAreaEmphasis
log-sigma-1-0-mm-3D_glszm_LargeAreaHighGrayLevelEmphasis
log-sigma-1-0-mm-3D_glszm_LargeAreaLowGrayLevelEmphasis
log-sigma-1-0-mm-3D_glszm_LowGrayLevelZoneEmphasis
log-sigma-1-0-mm-3D_glszm_SizeZoneNonUniformity
log-sigma-1-0-mm-3D_glszm_SizeZoneNonUniformityNormalized
log-sigma-1-0-mm-3D_glszm_SmallAreaEmphasis
log-sigma-1-0-mm-3D_glszm_SmallAreaHighGrayLevelEmphasis
log-sigma-1-0-mm-3D_glszm_SmallAreaLowGrayLevelEmphasis
log-sigma-1-0-mm-3D_glszm_ZoneEntropy
log-sigma-1-0-mm-3D_glszm_ZonePercentage
log-sigma-1-0-mm-3D_glszm_ZoneVariance
log-sigma-1-0-mm-3D_ngtdm_Busyness
log-sigma-1-0-mm-3D_ngtdm_Coarseness
log-sigma-1-0-mm-3D_ngtdm_Complexity
log-sigma-1-0-mm-3D_ngtdm_Contrast
log-sigma-1-0-mm-3D_ngtdm_Strength
log-sigma-2-0-mm-3D_firstorder_10Percentile
log-sigma-2-0-mm-3D_firstorder_90Percentile
log-sigma-2-0-mm-3D_firstorder_Energy
log-sigma-2-0-mm-3D_firstorder_Entropy
log-sigma-2-0-mm-3D_firstorder_InterquartileRange
log-sigma-2-0-mm-3D_firstorder_Kurtosis
log-sigma-2-0-mm-3D_firstorder_Maximum
log-sigma-2-0-mm-3D_firstorder_MeanAbsoluteDeviation
log-sigma-2-0-mm-3D_firstorder_Mean
log-sigma-2-0-mm-3D_firstorder_Median
log-sigma-2-0-mm-3D_firstorder_Minimum
log-sigma-2-0-mm-3D_firstorder_Range
log-sigma-2-0-mm-3D_firstorder_RobustMeanAbsoluteDeviation
log-sigma-2-0-mm-3D_firstorder_RootMeanSquared
log-sigma-2-0-mm-3D_firstorder_Skewness
log-sigma-2-0-mm-3D_firstorder_TotalEnergy
log-sigma-2-0-mm-3D_firstorder_Uniformity
log-sigma-2-0-mm-3D_firstorder_Variance

log-sigma-2-0-mm-3D_glcm_Autocorrelation
log-sigma-2-0-mm-3D_glcm_ClusterProminence
log-sigma-2-0-mm-3D_glcm_ClusterShade
log-sigma-2-0-mm-3D_glcm_ClusterTendency
log-sigma-2-0-mm-3D_glcm_Contrast
log-sigma-2-0-mm-3D_glcm_Correlation
log-sigma-2-0-mm-3D_glcm_DifferenceAverage
log-sigma-2-0-mm-3D_glcm_DifferenceEntropy
log-sigma-2-0-mm-3D_glcm_DifferenceVariance
log-sigma-2-0-mm-3D_glcm_Id
log-sigma-2-0-mm-3D_glcm_Idm
log-sigma-2-0-mm-3D_glcm_Idmn
log-sigma-2-0-mm-3D_glcm_Idn
log-sigma-2-0-mm-3D_glcm_Imc1
log-sigma-2-0-mm-3D_glcm_Imc2
log-sigma-2-0-mm-3D_glcm_InverseVariance
log-sigma-2-0-mm-3D_glcm_JointAverage
log-sigma-2-0-mm-3D_glcm_JointEnergy
log-sigma-2-0-mm-3D_glcm_JointEntropy
log-sigma-2-0-mm-3D_glcm_MCC
log-sigma-2-0-mm-3D_glcm_MaximumProbability
log-sigma-2-0-mm-3D_glcm_SumAverage
log-sigma-2-0-mm-3D_glcm_SumEntropy
log-sigma-2-0-mm-3D_glcm_SumSquares
log-sigma-2-0-mm-3D_gldm_DependenceEntropy
log-sigma-2-0-mm-3D_gldm_DependenceNonUniformity
log-sigma-2-0-mm-3D_gldm_DependenceNonUniformityNormalized
log-sigma-2-0-mm-3D_gldm_DependenceVariance
log-sigma-2-0-mm-3D_gldm_GrayLevelNonUniformity
log-sigma-2-0-mm-3D_gldm_GrayLevelVariance
log-sigma-2-0-mm-3D_gldm_HighGrayLevelEmphasis
log-sigma-2-0-mm-3D_gldm_LargeDependenceEmphasis
log-sigma-2-0-mm-3D_gldm_LargeDependenceHighGrayLevelEmphasis
log-sigma-2-0-mm-3D_gldm_LargeDependenceLowGrayLevelEmphasis
log-sigma-2-0-mm-3D_gldm_LowGrayLevelEmphasis
log-sigma-2-0-mm-3D_gldm_SmallDependenceEmphasis
log-sigma-2-0-mm-3D_gldm_SmallDependenceHighGrayLevelEmphasis
log-sigma-2-0-mm-3D_gldm_SmallDependenceLowGrayLevelEmphasis
log-sigma-2-0-mm-3D_glrlm_GrayLevelNonUniformity
log-sigma-2-0-mm-3D_glrlm_GrayLevelNonUniformityNormalized
log-sigma-2-0-mm-3D_glrlm_GrayLevelVariance
log-sigma-2-0-mm-3D_glrlm_HighGrayLevelRunEmphasis
log-sigma-2-0-mm-3D_glrlm_LongRunEmphasis
log-sigma-2-0-mm-3D_glrlm_LongRunHighGrayLevelEmphasis
log-sigma-2-0-mm-3D_glrlm_LongRunLowGrayLevelEmphasis
log-sigma-2-0-mm-3D_glrlm_LowGrayLevelRunEmphasis
log-sigma-2-0-mm-3D_glrlm_RunEntropy
log-sigma-2-0-mm-3D_glrlm_RunLengthNonUniformity
log-sigma-2-0-mm-3D_glrlm_RunLengthNonUniformityNormalized
log-sigma-2-0-mm-3D_glrlm_RunPercentage
log-sigma-2-0-mm-3D_glrlm_RunVariance
log-sigma-2-0-mm-3D_glrlm_ShortRunEmphasis
log-sigma-2-0-mm-3D_glrlm_ShortRunHighGrayLevelEmphasis

log-sigma-2-0-mm-3D_glrlm_ShortRunLowGrayLevelEmphasis
log-sigma-2-0-mm-3D_glszm_GrayLevelNonUniformity
log-sigma-2-0-mm-3D_glszm_GrayLevelNonUniformityNormalized
log-sigma-2-0-mm-3D_glszm_GrayLevelVariance
log-sigma-2-0-mm-3D_glszm_HighGrayLevelZoneEmphasis
log-sigma-2-0-mm-3D_glszm_LargeAreaEmphasis
log-sigma-2-0-mm-3D_glszm_LargeAreaHighGrayLevelEmphasis
log-sigma-2-0-mm-3D_glszm_LargeAreaLowGrayLevelEmphasis
log-sigma-2-0-mm-3D_glszm_LowGrayLevelZoneEmphasis
log-sigma-2-0-mm-3D_glszm_SizeZoneNonUniformity
log-sigma-2-0-mm-3D_glszm_SizeZoneNonUniformityNormalized
log-sigma-2-0-mm-3D_glszm_SmallAreaEmphasis
log-sigma-2-0-mm-3D_glszm_SmallAreaHighGrayLevelEmphasis
log-sigma-2-0-mm-3D_glszm_SmallAreaLowGrayLevelEmphasis
log-sigma-2-0-mm-3D_glszm_ZoneEntropy
log-sigma-2-0-mm-3D_glszm_ZonePercentage
log-sigma-2-0-mm-3D_glszm_ZoneVariance
log-sigma-2-0-mm-3D_ngtdm_Busyness
log-sigma-2-0-mm-3D_ngtdm_Coarseness
log-sigma-2-0-mm-3D_ngtdm_Complexity
log-sigma-2-0-mm-3D_ngtdm_Contrast
log-sigma-2-0-mm-3D_ngtdm_Strength
log-sigma-5-0-mm-3D_firstorder_10Percentile
log-sigma-5-0-mm-3D_firstorder_90Percentile
log-sigma-5-0-mm-3D_firstorder_Energy
log-sigma-5-0-mm-3D_firstorder_Entropy
log-sigma-5-0-mm-3D_firstorder_InterquartileRange
log-sigma-5-0-mm-3D_firstorder_Kurtosis
log-sigma-5-0-mm-3D_firstorder_Maximum
log-sigma-5-0-mm-3D_firstorder_MeanAbsoluteDeviation
log-sigma-5-0-mm-3D_firstorder_Mean
log-sigma-5-0-mm-3D_firstorder_Median
log-sigma-5-0-mm-3D_firstorder_Minimum
log-sigma-5-0-mm-3D_firstorder_Range
log-sigma-5-0-mm-3D_firstorder_RobustMeanAbsoluteDeviation
log-sigma-5-0-mm-3D_firstorder_RootMeanSquared
log-sigma-5-0-mm-3D_firstorder_Skewness
log-sigma-5-0-mm-3D_firstorder_TotalEnergy
log-sigma-5-0-mm-3D_firstorder_Uniformity
log-sigma-5-0-mm-3D_firstorder_Variance
log-sigma-5-0-mm-3D_glcm_Autocorrelation
log-sigma-5-0-mm-3D_glcm_ClusterProminence
log-sigma-5-0-mm-3D_glcm_ClusterShade
log-sigma-5-0-mm-3D_glcm_ClusterTendency
log-sigma-5-0-mm-3D_glcm_Contrast
log-sigma-5-0-mm-3D_glcm_Correlation
log-sigma-5-0-mm-3D_glcm_DifferenceAverage
log-sigma-5-0-mm-3D_glcm_DifferenceEntropy
log-sigma-5-0-mm-3D_glcm_DifferenceVariance
log-sigma-5-0-mm-3D_glcm_Id
log-sigma-5-0-mm-3D_glcm_Idm
log-sigma-5-0-mm-3D_glcm_Idmn
log-sigma-5-0-mm-3D_glcm_Idn

log-sigma-5-0-mm-3D_glcm_Imc1
log-sigma-5-0-mm-3D_glcm_Imc2
log-sigma-5-0-mm-3D_glcm_InverseVariance
log-sigma-5-0-mm-3D_glcm_JointAverage
log-sigma-5-0-mm-3D_glcm_JointEnergy
log-sigma-5-0-mm-3D_glcm_JointEntropy
log-sigma-5-0-mm-3D_glcm_MCC
log-sigma-5-0-mm-3D_glcm_MaximumProbability
log-sigma-5-0-mm-3D_glcm_SumAverage
log-sigma-5-0-mm-3D_glcm_SumEntropy
log-sigma-5-0-mm-3D_glcm_SumSquares
log-sigma-5-0-mm-3D_gldm_DependenceEntropy
log-sigma-5-0-mm-3D_gldm_DependenceNonUniformity
log-sigma-5-0-mm-3D_gldm_DependenceNonUniformityNormalized
log-sigma-5-0-mm-3D_gldm_DependenceVariance
log-sigma-5-0-mm-3D_gldm_GrayLevelNonUniformity
log-sigma-5-0-mm-3D_gldm_GrayLevelVariance
log-sigma-5-0-mm-3D_gldm_HighGrayLevelEmphasis
log-sigma-5-0-mm-3D_gldm_LargeDependenceEmphasis
log-sigma-5-0-mm-3D_gldm_LargeDependenceHighGrayLevelEmphasis
log-sigma-5-0-mm-3D_gldm_LargeDependenceLowGrayLevelEmphasis
log-sigma-5-0-mm-3D_gldm_LowGrayLevelEmphasis
log-sigma-5-0-mm-3D_gldm_SmallDependenceEmphasis
log-sigma-5-0-mm-3D_gldm_SmallDependenceHighGrayLevelEmphasis
log-sigma-5-0-mm-3D_gldm_SmallDependenceLowGrayLevelEmphasis
log-sigma-5-0-mm-3D_glrlm_GrayLevelNonUniformity
log-sigma-5-0-mm-3D_glrlm_GrayLevelNonUniformityNormalized
log-sigma-5-0-mm-3D_glrlm_GrayLevelVariance
log-sigma-5-0-mm-3D_glrlm_HighGrayLevelRunEmphasis
log-sigma-5-0-mm-3D_glrlm_LongRunEmphasis
log-sigma-5-0-mm-3D_glrlm_LongRunHighGrayLevelEmphasis
log-sigma-5-0-mm-3D_glrlm_LongRunLowGrayLevelEmphasis
log-sigma-5-0-mm-3D_glrlm_LowGrayLevelRunEmphasis
log-sigma-5-0-mm-3D_glrlm_RunEntropy
log-sigma-5-0-mm-3D_glrlm_RunLengthNonUniformity
log-sigma-5-0-mm-3D_glrlm_RunLengthNonUniformityNormalized
log-sigma-5-0-mm-3D_glrlm_RunPercentage
log-sigma-5-0-mm-3D_glrlm_RunVariance
log-sigma-5-0-mm-3D_glrlm_ShortRunEmphasis
log-sigma-5-0-mm-3D_glrlm_ShortRunHighGrayLevelEmphasis
log-sigma-5-0-mm-3D_glrlm_ShortRunLowGrayLevelEmphasis
log-sigma-5-0-mm-3D_glszm_GrayLevelNonUniformity
log-sigma-5-0-mm-3D_glszm_GrayLevelNonUniformityNormalized
log-sigma-5-0-mm-3D_glszm_GrayLevelVariance
log-sigma-5-0-mm-3D_glszm_HighGrayLevelZoneEmphasis
log-sigma-5-0-mm-3D_glszm_LargeAreaEmphasis
log-sigma-5-0-mm-3D_glszm_LargeAreaHighGrayLevelEmphasis
log-sigma-5-0-mm-3D_glszm_LargeAreaLowGrayLevelEmphasis
log-sigma-5-0-mm-3D_glszm_LowGrayLevelZoneEmphasis
log-sigma-5-0-mm-3D_glszm_SizeZoneNonUniformity
log-sigma-5-0-mm-3D_glszm_SizeZoneNonUniformityNormalized
log-sigma-5-0-mm-3D_glszm_SmallAreaEmphasis
log-sigma-5-0-mm-3D_glszm_SmallAreaHighGrayLevelEmphasis

log-sigma-5-0-mm-3D_glszm_SmallAreaLowGrayLevelEmphasis
log-sigma-5-0-mm-3D_glszm_ZoneEntropy
log-sigma-5-0-mm-3D_glszm_ZonePercentage
log-sigma-5-0-mm-3D_glszm_ZoneVariance
log-sigma-5-0-mm-3D_ngtdm_Busyness
log-sigma-5-0-mm-3D_ngtdm_Coarseness
log-sigma-5-0-mm-3D_ngtdm_Complexity
log-sigma-5-0-mm-3D_ngtdm_Contrast
log-sigma-5-0-mm-3D_ngtdm_Strength
logarithm_firstorder_10Percentile
logarithm_firstorder_90Percentile
logarithm_firstorder_Energy
logarithm_firstorder_Entropy
logarithm_firstorder_InterquartileRange
logarithm_firstorder_Kurtosis
logarithm_firstorder_Maximum
logarithm_firstorder_MeanAbsoluteDeviation
logarithm_firstorder_Mean
logarithm_firstorder_Median
logarithm_firstorder_Minimum
logarithm_firstorder_Range
logarithm_firstorder_RobustMeanAbsoluteDeviation
logarithm_firstorder_RootMeanSquared
logarithm_firstorder_Skewness
logarithm_firstorder_TotalEnergy
logarithm_firstorder_Uniformity
logarithm_firstorder_Variance
logarithm_glcm_Autocorrelation
logarithm_glcm_ClusterProminence
logarithm_glcm_ClusterShade
logarithm_glcm_ClusterTendency
logarithm_glcm_Contrast
logarithm_glcm_Correlation
logarithm_glcm_DifferenceAverage
logarithm_glcm_DifferenceEntropy
logarithm_glcm_DifferenceVariance
logarithm_glcm_Id
logarithm_glcm_Idm
logarithm_glcm_Idmn
logarithm_glcm_Idn
logarithm_glcm_Imc1
logarithm_glcm_Imc2
logarithm_glcm_InverseVariance
logarithm_glcm_JointAverage
logarithm_glcm_JointEnergy
logarithm_glcm_JointEntropy
logarithm_glcm_MCC
logarithm_glcm_MaximumProbability
logarithm_glcm_SumAverage
logarithm_glcm_SumEntropy
logarithm_glcm_SumSquares
logarithm_gldm_DependenceEntropy
logarithm_gldm_DependenceNonUniformity

logarithm_gldm_DependenceNonUniformityNormalized
logarithm_gldm_DependenceVariance
logarithm_gldm_GrayLevelNonUniformity
logarithm_gldm_GrayLevelVariance
logarithm_gldm_HighGrayLevelEmphasis
logarithm_gldm_LargeDependenceEmphasis
logarithm_gldm_LargeDependenceHighGrayLevelEmphasis
logarithm_gldm_LargeDependenceLowGrayLevelEmphasis
logarithm_gldm_LowGrayLevelEmphasis
logarithm_gldm_SmallDependenceEmphasis
logarithm_gldm_SmallDependenceHighGrayLevelEmphasis
logarithm_gldm_SmallDependenceLowGrayLevelEmphasis
logarithm_glrlm_GrayLevelNonUniformity
logarithm_glrlm_GrayLevelNonUniformityNormalized
logarithm_glrlm_GrayLevelVariance
logarithm_glrlm_HighGrayLevelRunEmphasis
logarithm_glrlm_LongRunEmphasis
logarithm_glrlm_LongRunHighGrayLevelEmphasis
logarithm_glrlm_LongRunLowGrayLevelEmphasis
logarithm_glrlm_LowGrayLevelRunEmphasis
logarithm_glrlm_RunEntropy
logarithm_glrlm_RunLengthNonUniformity
logarithm_glrlm_RunLengthNonUniformityNormalized
logarithm_glrlm_RunPercentage
logarithm_glrlm_RunVariance
logarithm_glrlm_ShortRunEmphasis
logarithm_glrlm_ShortRunHighGrayLevelEmphasis
logarithm_glrlm_ShortRunLowGrayLevelEmphasis
logarithm_glszm_GrayLevelNonUniformity
logarithm_glszm_GrayLevelNonUniformityNormalized
logarithm_glszm_GrayLevelVariance
logarithm_glszm_HighGrayLevelZoneEmphasis
logarithm_glszm_LargeAreaEmphasis
logarithm_glszm_LargeAreaHighGrayLevelEmphasis
logarithm_glszm_LargeAreaLowGrayLevelEmphasis
logarithm_glszm_LowGrayLevelZoneEmphasis
logarithm_glszm_SizeZoneNonUniformity
logarithm_glszm_SizeZoneNonUniformityNormalized
logarithm_glszm_SmallAreaEmphasis
logarithm_glszm_SmallAreaHighGrayLevelEmphasis
logarithm_glszm_SmallAreaLowGrayLevelEmphasis
logarithm_glszm_ZoneEntropy
logarithm_glszm_ZonePercentage
logarithm_glszm_ZoneVariance
logarithm_ngtdm_Busyness
logarithm_ngtdm_Coarseness
logarithm_ngtdm_Complexity
logarithm_ngtdm_Contrast
logarithm_ngtdm_Strength
square_firstorder_10Percentile
square_firstorder_90Percentile
square_firstorder_Energy
square_firstorder_Entropy

square_firstorder_InterquartileRange
square_firstorder_Kurtosis
square_firstorder_Maximum
square_firstorder_MeanAbsoluteDeviation
square_firstorder_Mean
square_firstorder_Median
square_firstorder_Minimum
square_firstorder_Range
square_firstorder_RobustMeanAbsoluteDeviation
square_firstorder_RootMeanSquared
square_firstorder_Skewness
square_firstorder_TotalEnergy
square_firstorder_Uniformity
square_firstorder_Variance
square_glcm_Autocorrelation
square_glcm_ClusterProminence
square_glcm_ClusterShade
square_glcm_ClusterTendency
square_glcm_Contrast
square_glcm_Correlation
square_glcm_DifferenceAverage
square_glcm_DifferenceEntropy
square_glcm_DifferenceVariance
square_glcm_Id
square_glcm_Idm
square_glcm_Idmn
square_glcm_Idn
square_glcm_Imc1
square_glcm_Imc2
square_glcm_InverseVariance
square_glcm_JointAverage
square_glcm_JointEnergy
square_glcm_JointEntropy
square_glcm_MCC
square_glcm_MaximumProbability
square_glcm_SumAverage
square_glcm_SumEntropy
square_glcm_SumSquares
square_gldm_DependenceEntropy
square_gldm_DependenceNonUniformity
square_gldm_DependenceNonUniformityNormalized
square_gldm_DependenceVariance
square_gldm_GrayLevelNonUniformity
square_gldm_GrayLevelVariance
square_gldm_HighGrayLevelEmphasis
square_gldm_LargeDependenceEmphasis
square_gldm_LargeDependenceHighGrayLevelEmphasis
square_gldm_LargeDependenceLowGrayLevelEmphasis
square_gldm_LowGrayLevelEmphasis
square_gldm_SmallDependenceEmphasis
square_gldm_SmallDependenceHighGrayLevelEmphasis
square_gldm_SmallDependenceLowGrayLevelEmphasis
square_glrlm_GrayLevelNonUniformity

square_glrlm_GrayLevelNonUniformityNormalized
square_glrlm_GrayLevelVariance
square_glrlm_HighGrayLevelRunEmphasis
square_glrlm_LongRunEmphasis
square_glrlm_LongRunHighGrayLevelEmphasis
square_glrlm_LongRunLowGrayLevelEmphasis
square_glrlm_LowGrayLevelRunEmphasis
square_glrlm_RunEntropy
square_glrlm_RunLengthNonUniformity
square_glrlm_RunLengthNonUniformityNormalized
square_glrlm_RunPercentage
square_glrlm_RunVariance
square_glrlm_ShortRunEmphasis
square_glrlm_ShortRunHighGrayLevelEmphasis
square_glrlm_ShortRunLowGrayLevelEmphasis
square_glszm_GrayLevelNonUniformity
square_glszm_GrayLevelNonUniformityNormalized
square_glszm_GrayLevelVariance
square_glszm_HighGrayLevelZoneEmphasis
square_glszm_LargeAreaEmphasis
square_glszm_LargeAreaHighGrayLevelEmphasis
square_glszm_LargeAreaLowGrayLevelEmphasis
square_glszm_LowGrayLevelZoneEmphasis
square_glszm_SizeZoneNonUniformity
square_glszm_SizeZoneNonUniformityNormalized
square_glszm_SmallAreaEmphasis
square_glszm_SmallAreaHighGrayLevelEmphasis
square_glszm_SmallAreaLowGrayLevelEmphasis
square_glszm_ZoneEntropy
square_glszm_ZonePercentage
square_glszm_ZoneVariance
square_ngtdm_Busyness
square_ngtdm_Coarseness
square_ngtdm_Complexity
square_ngtdm_Contrast
square_ngtdm_Strength
squareroot_firstorder_10Percentile
squareroot_firstorder_90Percentile
squareroot_firstorder_Energy
squareroot_firstorder_Entropy
squareroot_firstorder_InterquartileRange
squareroot_firstorder_Kurtosis
squareroot_firstorder_Maximum
squareroot_firstorder_MeanAbsoluteDeviation
squareroot_firstorder_Mean
squareroot_firstorder_Median
squareroot_firstorder_Minimum
squareroot_firstorder_Range
squareroot_firstorder_RobustMeanAbsoluteDeviation
squareroot_firstorder_RootMeanSquared
squareroot_firstorder_Skewness
squareroot_firstorder_TotalEnergy
squareroot_firstorder_Uniformity

squareroot_firstorder_Variance
squareroot_glcm_Autocorrelation
squareroot_glcm_ClusterProminence
squareroot_glcm_ClusterShade
squareroot_glcm_ClusterTendency
squareroot_glcm_Contrast
squareroot_glcm_Correlation
squareroot_glcm_DifferenceAverage
squareroot_glcm_DifferenceEntropy
squareroot_glcm_DifferenceVariance
squareroot_glcm_Id
squareroot_glcm_Idm
squareroot_glcm_Idmn
squareroot_glcm_Idn
squareroot_glcm_Imc1
squareroot_glcm_Imc2
squareroot_glcm_InverseVariance
squareroot_glcm_JointAverage
squareroot_glcm_JointEnergy
squareroot_glcm_JointEntropy
squareroot_glcm_MCC
squareroot_glcm_MaximumProbability
squareroot_glcm_SumAverage
squareroot_glcm_SumEntropy
squareroot_glcm_SumSquares
squareroot_gldm_DependenceEntropy
squareroot_gldm_DependenceNonUniformity
squareroot_gldm_DependenceNonUniformityNormalized
squareroot_gldm_DependenceVariance
squareroot_gldm_GrayLevelNonUniformity
squareroot_gldm_GrayLevelVariance
squareroot_gldm_HighGrayLevelEmphasis
squareroot_gldm_LargeDependenceEmphasis
squareroot_gldm_LargeDependenceHighGrayLevelEmphasis
squareroot_gldm_LargeDependenceLowGrayLevelEmphasis
squareroot_gldm_LowGrayLevelEmphasis
squareroot_gldm_SmallDependenceEmphasis
squareroot_gldm_SmallDependenceHighGrayLevelEmphasis
squareroot_gldm_SmallDependenceLowGrayLevelEmphasis
squareroot_glrlm_GrayLevelNonUniformity
squareroot_glrlm_GrayLevelNonUniformityNormalized
squareroot_glrlm_GrayLevelVariance
squareroot_glrlm_HighGrayLevelRunEmphasis
squareroot_glrlm_LongRunEmphasis
squareroot_glrlm_LongRunHighGrayLevelEmphasis
squareroot_glrlm_LongRunLowGrayLevelEmphasis
squareroot_glrlm_LowGrayLevelRunEmphasis
squareroot_glrlm_RunEntropy
squareroot_glrlm_RunLengthNonUniformity
squareroot_glrlm_RunLengthNonUniformityNormalized
squareroot_glrlm_RunPercentage
squareroot_glrlm_RunVariance
squareroot_glrlm_ShortRunEmphasis

squareroot_glrlm_ShortRunHighGrayLevelEmphasis
squareroot_glrlm_ShortRunLowGrayLevelEmphasis
squareroot_glszm_GrayLevelNonUniformity
squareroot_glszm_GrayLevelNonUniformityNormalized
squareroot_glszm_GrayLevelVariance
squareroot_glszm_HighGrayLevelZoneEmphasis
squareroot_glszm_LargeAreaEmphasis
squareroot_glszm_LargeAreaHighGrayLevelEmphasis
squareroot_glszm_LargeAreaLowGrayLevelEmphasis
squareroot_glszm_LowGrayLevelZoneEmphasis
squareroot_glszm_SizeZoneNonUniformity
squareroot_glszm_SizeZoneNonUniformityNormalized
squareroot_glszm_SmallAreaEmphasis
squareroot_glszm_SmallAreaHighGrayLevelEmphasis
squareroot_glszm_SmallAreaLowGrayLevelEmphasis
squareroot_glszm_ZoneEntropy
squareroot_glszm_ZonePercentage
squareroot_glszm_ZoneVariance
squareroot_ngtdm_Busyness
squareroot_ngtdm_Coarseness
squareroot_ngtdm_Complexity
squareroot_ngtdm_Contrast
squareroot_ngtdm_Strength
wavelet-LLH_firstorder_10Percentile
wavelet-LLH_firstorder_90Percentile
wavelet-LLH_firstorder_Energy
wavelet-LLH_firstorder_Entropy
wavelet-LLH_firstorder_InterquartileRange
wavelet-LLH_firstorder_Kurtosis
wavelet-LLH_firstorder_Maximum
wavelet-LLH_firstorder_MeanAbsoluteDeviation
wavelet-LLH_firstorder_Mean
wavelet-LLH_firstorder_Median
wavelet-LLH_firstorder_Minimum
wavelet-LLH_firstorder_Range
wavelet-LLH_firstorder_RobustMeanAbsoluteDeviation
wavelet-LLH_firstorder_RootMeanSquared
wavelet-LLH_firstorder_Skewness
wavelet-LLH_firstorder_TotalEnergy
wavelet-LLH_firstorder_Uniformity
wavelet-LLH_firstorder_Variance
wavelet-LLH_glcm_Autocorrelation
wavelet-LLH_glcm_ClusterProminence
wavelet-LLH_glcm_ClusterShade
wavelet-LLH_glcm_ClusterTendency
wavelet-LLH_glcm_Contrast
wavelet-LLH_glcm_Correlation
wavelet-LLH_glcm_DifferenceAverage
wavelet-LLH_glcm_DifferenceEntropy
wavelet-LLH_glcm_DifferenceVariance
wavelet-LLH_glcm_Id
wavelet-LLH_glcm_Idm
wavelet-LLH_glcm_Idmn

wavelet-LLH_glcm_Idn
wavelet-LLH_glcm_Imc1
wavelet-LLH_glcm_Imc2
wavelet-LLH_glcm_InverseVariance
wavelet-LLH_glcm_JointAverage
wavelet-LLH_glcm_JointEnergy
wavelet-LLH_glcm_JointEntropy
wavelet-LLH_glcm_MCC
wavelet-LLH_glcm_MaximumProbability
wavelet-LLH_glcm_SumAverage
wavelet-LLH_glcm_SumEntropy
wavelet-LLH_glcm_SumSquares
wavelet-LLH_gldm_DependenceEntropy
wavelet-LLH_gldm_DependenceNonUniformity
wavelet-LLH_gldm_DependenceNonUniformityNormalized
wavelet-LLH_gldm_DependenceVariance
wavelet-LLH_gldm_GrayLevelNonUniformity
wavelet-LLH_gldm_GrayLevelVariance
wavelet-LLH_gldm_HighGrayLevelEmphasis
wavelet-LLH_gldm_LargeDependenceEmphasis
wavelet-LLH_gldm_LargeDependenceHighGrayLevelEmphasis
wavelet-LLH_gldm_LargeDependenceLowGrayLevelEmphasis
wavelet-LLH_gldm_LowGrayLevelEmphasis
wavelet-LLH_gldm_SmallDependenceEmphasis
wavelet-LLH_gldm_SmallDependenceHighGrayLevelEmphasis
wavelet-LLH_gldm_SmallDependenceLowGrayLevelEmphasis
wavelet-LLH_glrlm_GrayLevelNonUniformity
wavelet-LLH_glrlm_GrayLevelNonUniformityNormalized
wavelet-LLH_glrlm_GrayLevelVariance
wavelet-LLH_glrlm_HighGrayLevelRunEmphasis
wavelet-LLH_glrlm_LongRunEmphasis
wavelet-LLH_glrlm_LongRunHighGrayLevelEmphasis
wavelet-LLH_glrlm_LongRunLowGrayLevelEmphasis
wavelet-LLH_glrlm_LowGrayLevelRunEmphasis
wavelet-LLH_glrlm_RunEntropy
wavelet-LLH_glrlm_RunLengthNonUniformity
wavelet-LLH_glrlm_RunLengthNonUniformityNormalized
wavelet-LLH_glrlm_RunPercentage
wavelet-LLH_glrlm_RunVariance
wavelet-LLH_glrlm_ShortRunEmphasis
wavelet-LLH_glrlm_ShortRunHighGrayLevelEmphasis
wavelet-LLH_glrlm_ShortRunLowGrayLevelEmphasis
wavelet-LLH_glszm_GrayLevelNonUniformity
wavelet-LLH_glszm_GrayLevelNonUniformityNormalized
wavelet-LLH_glszm_GrayLevelVariance
wavelet-LLH_glszm_HighGrayLevelZoneEmphasis
wavelet-LLH_glszm_LargeAreaEmphasis
wavelet-LLH_glszm_LargeAreaHighGrayLevelEmphasis
wavelet-LLH_glszm_LargeAreaLowGrayLevelEmphasis
wavelet-LLH_glszm_LowGrayLevelZoneEmphasis
wavelet-LLH_glszm_SizeZoneNonUniformity
wavelet-LLH_glszm_SizeZoneNonUniformityNormalized
wavelet-LLH_glszm_SmallAreaEmphasis

wavelet-LLH_glszm_SmallAreaHighGrayLevelEmphasis
wavelet-LLH_glszm_SmallAreaLowGrayLevelEmphasis
wavelet-LLH_glszm_ZoneEntropy
wavelet-LLH_glszm_ZonePercentage
wavelet-LLH_glszm_ZoneVariance
wavelet-LLH_ngtdm_Busyness
wavelet-LLH_ngtdm_Coarseness
wavelet-LLH_ngtdm_Complexity
wavelet-LLH_ngtdm_Contrast
wavelet-LLH_ngtdm_Strength
wavelet-LHL_firstorder_10Percentile
wavelet-LHL_firstorder_90Percentile
wavelet-LHL_firstorder_Energy
wavelet-LHL_firstorder_Entropy
wavelet-LHL_firstorder_InterquartileRange
wavelet-LHL_firstorder_Kurtosis
wavelet-LHL_firstorder_Maximum
wavelet-LHL_firstorder_MeanAbsoluteDeviation
wavelet-LHL_firstorder_Mean
wavelet-LHL_firstorder_Median
wavelet-LHL_firstorder_Minimum
wavelet-LHL_firstorder_Range
wavelet-LHL_firstorder_RobustMeanAbsoluteDeviation
wavelet-LHL_firstorder_RootMeanSquared
wavelet-LHL_firstorder_Skewness
wavelet-LHL_firstorder_TotalEnergy
wavelet-LHL_firstorder_Uniformity
wavelet-LHL_firstorder_Variance
wavelet-LHL_glcm_Autocorrelation
wavelet-LHL_glcm_ClusterProminence
wavelet-LHL_glcm_ClusterShade
wavelet-LHL_glcm_ClusterTendency
wavelet-LHL_glcm_Contrast
wavelet-LHL_glcm_Correlation
wavelet-LHL_glcm_DifferenceAverage
wavelet-LHL_glcm_DifferenceEntropy
wavelet-LHL_glcm_DifferenceVariance
wavelet-LHL_glcm_Id
wavelet-LHL_glcm_Idm
wavelet-LHL_glcm_Idmn
wavelet-LHL_glcm_Idn
wavelet-LHL_glcm_Imc1
wavelet-LHL_glcm_Imc2
wavelet-LHL_glcm_InverseVariance
wavelet-LHL_glcm_JointAverage
wavelet-LHL_glcm_JointEnergy
wavelet-LHL_glcm_JointEntropy
wavelet-LHL_glcm_MCC
wavelet-LHL_glcm_MaximumProbability
wavelet-LHL_glcm_SumAverage
wavelet-LHL_glcm_SumEntropy
wavelet-LHL_glcm_SumSquares
wavelet-LHL_gldm_DependenceEntropy

wavelet-LHL_gldm_DependenceNonUniformity
wavelet-LHL_gldm_DependenceNonUniformityNormalized
wavelet-LHL_gldm_DependenceVariance
wavelet-LHL_gldm_GrayLevelNonUniformity
wavelet-LHL_gldm_GrayLevelVariance
wavelet-LHL_gldm_HighGrayLevelEmphasis
wavelet-LHL_gldm_LargeDependenceEmphasis
wavelet-LHL_gldm_LargeDependenceHighGrayLevelEmphasis
wavelet-LHL_gldm_LargeDependenceLowGrayLevelEmphasis
wavelet-LHL_gldm_LowGrayLevelEmphasis
wavelet-LHL_gldm_SmallDependenceEmphasis
wavelet-LHL_gldm_SmallDependenceHighGrayLevelEmphasis
wavelet-LHL_gldm_SmallDependenceLowGrayLevelEmphasis
wavelet-LHL_glrlm_GrayLevelNonUniformity
wavelet-LHL_glrlm_GrayLevelNonUniformityNormalized
wavelet-LHL_glrlm_GrayLevelVariance
wavelet-LHL_glrlm_HighGrayLevelRunEmphasis
wavelet-LHL_glrlm_LongRunEmphasis
wavelet-LHL_glrlm_LongRunHighGrayLevelEmphasis
wavelet-LHL_glrlm_LongRunLowGrayLevelEmphasis
wavelet-LHL_glrlm_LowGrayLevelRunEmphasis
wavelet-LHL_glrlm_RunEntropy
wavelet-LHL_glrlm_RunLengthNonUniformity
wavelet-LHL_glrlm_RunLengthNonUniformityNormalized
wavelet-LHL_glrlm_RunPercentage
wavelet-LHL_glrlm_RunVariance
wavelet-LHL_glrlm_ShortRunEmphasis
wavelet-LHL_glrlm_ShortRunHighGrayLevelEmphasis
wavelet-LHL_glrlm_ShortRunLowGrayLevelEmphasis
wavelet-LHL_glszm_GrayLevelNonUniformity
wavelet-LHL_glszm_GrayLevelNonUniformityNormalized
wavelet-LHL_glszm_GrayLevelVariance
wavelet-LHL_glszm_HighGrayLevelZoneEmphasis
wavelet-LHL_glszm_LargeAreaEmphasis
wavelet-LHL_glszm_LargeAreaHighGrayLevelEmphasis
wavelet-LHL_glszm_LargeAreaLowGrayLevelEmphasis
wavelet-LHL_glszm_LowGrayLevelZoneEmphasis
wavelet-LHL_glszm_SizeZoneNonUniformity
wavelet-LHL_glszm_SizeZoneNonUniformityNormalized
wavelet-LHL_glszm_SmallAreaEmphasis
wavelet-LHL_glszm_SmallAreaHighGrayLevelEmphasis
wavelet-LHL_glszm_SmallAreaLowGrayLevelEmphasis
wavelet-LHL_glszm_ZoneEntropy
wavelet-LHL_glszm_ZonePercentage
wavelet-LHL_glszm_ZoneVariance
wavelet-LHL_ngtdm_Busyness
wavelet-LHL_ngtdm_Coarseness
wavelet-LHL_ngtdm_Complexity
wavelet-LHL_ngtdm_Contrast
wavelet-LHL_ngtdm_Strength
wavelet-LHH_firstorder_10Percentile
wavelet-LHH_firstorder_90Percentile
wavelet-LHH_firstorder_Energy

wavelet-LHH_firstorder_Entropy
wavelet-LHH_firstorder_InterquartileRange
wavelet-LHH_firstorder_Kurtosis
wavelet-LHH_firstorder_Maximum
wavelet-LHH_firstorder_MeanAbsoluteDeviation
wavelet-LHH_firstorder_Mean
wavelet-LHH_firstorder_Median
wavelet-LHH_firstorder_Minimum
wavelet-LHH_firstorder_Range
wavelet-LHH_firstorder_RobustMeanAbsoluteDeviation
wavelet-LHH_firstorder_RootMeanSquared
wavelet-LHH_firstorder_Skewness
wavelet-LHH_firstorder_TotalEnergy
wavelet-LHH_firstorder_Uniformity
wavelet-LHH_firstorder_Variance
wavelet-LHH_glcm_Autocorrelation
wavelet-LHH_glcm_ClusterProminence
wavelet-LHH_glcm_ClusterShade
wavelet-LHH_glcm_ClusterTendency
wavelet-LHH_glcm_Contrast
wavelet-LHH_glcm_Correlation
wavelet-LHH_glcm_DifferenceAverage
wavelet-LHH_glcm_DifferenceEntropy
wavelet-LHH_glcm_DifferenceVariance
wavelet-LHH_glcm_Id
wavelet-LHH_glcm_Idm
wavelet-LHH_glcm_Idmn
wavelet-LHH_glcm_Idn
wavelet-LHH_glcm_Imc1
wavelet-LHH_glcm_Imc2
wavelet-LHH_glcm_InverseVariance
wavelet-LHH_glcm_JointAverage
wavelet-LHH_glcm_JointEnergy
wavelet-LHH_glcm_JointEntropy
wavelet-LHH_glcm_MCC
wavelet-LHH_glcm_MaximumProbability
wavelet-LHH_glcm_SumAverage
wavelet-LHH_glcm_SumEntropy
wavelet-LHH_glcm_SumSquares
wavelet-LHH_gldm_DependenceEntropy
wavelet-LHH_gldm_DependenceNonUniformity
wavelet-LHH_gldm_DependenceNonUniformityNormalized
wavelet-LHH_gldm_DependenceVariance
wavelet-LHH_gldm_GrayLevelNonUniformity
wavelet-LHH_gldm_GrayLevelVariance
wavelet-LHH_gldm_HighGrayLevelEmphasis
wavelet-LHH_gldm_LargeDependenceEmphasis
wavelet-LHH_gldm_LargeDependenceHighGrayLevelEmphasis
wavelet-LHH_gldm_LargeDependenceLowGrayLevelEmphasis
wavelet-LHH_gldm_LowGrayLevelEmphasis
wavelet-LHH_gldm_SmallDependenceEmphasis
wavelet-LHH_gldm_SmallDependenceHighGrayLevelEmphasis
wavelet-LHH_gldm_SmallDependenceLowGrayLevelEmphasis

wavelet-LHH_glrlm_GrayLevelNonUniformity
wavelet-LHH_glrlm_GrayLevelNonUniformityNormalized
wavelet-LHH_glrlm_GrayLevelVariance
wavelet-LHH_glrlm_HighGrayLevelRunEmphasis
wavelet-LHH_glrlm_LongRunEmphasis
wavelet-LHH_glrlm_LongRunHighGrayLevelEmphasis
wavelet-LHH_glrlm_LongRunLowGrayLevelEmphasis
wavelet-LHH_glrlm_LowGrayLevelRunEmphasis
wavelet-LHH_glrlm_RunEntropy
wavelet-LHH_glrlm_RunLengthNonUniformity
wavelet-LHH_glrlm_RunLengthNonUniformityNormalized
wavelet-LHH_glrlm_RunPercentage
wavelet-LHH_glrlm_RunVariance
wavelet-LHH_glrlm_ShortRunEmphasis
wavelet-LHH_glrlm_ShortRunHighGrayLevelEmphasis
wavelet-LHH_glrlm_ShortRunLowGrayLevelEmphasis
wavelet-LHH_glszm_GrayLevelNonUniformity
wavelet-LHH_glszm_GrayLevelNonUniformityNormalized
wavelet-LHH_glszm_GrayLevelVariance
wavelet-LHH_glszm_HighGrayLevelZoneEmphasis
wavelet-LHH_glszm_LargeAreaEmphasis
wavelet-LHH_glszm_LargeAreaHighGrayLevelEmphasis
wavelet-LHH_glszm_LargeAreaLowGrayLevelEmphasis
wavelet-LHH_glszm_LowGrayLevelZoneEmphasis
wavelet-LHH_glszm_SizeZoneNonUniformity
wavelet-LHH_glszm_SizeZoneNonUniformityNormalized
wavelet-LHH_glszm_SmallAreaEmphasis
wavelet-LHH_glszm_SmallAreaHighGrayLevelEmphasis
wavelet-LHH_glszm_SmallAreaLowGrayLevelEmphasis
wavelet-LHH_glszm_ZoneEntropy
wavelet-LHH_glszm_ZonePercentage
wavelet-LHH_glszm_ZoneVariance
wavelet-LHH_ngtdm_Busyness
wavelet-LHH_ngtdm_Coarseness
wavelet-LHH_ngtdm_Complexity
wavelet-LHH_ngtdm_Contrast
wavelet-LHH_ngtdm_Strength
wavelet-HLL_firstorder_10Percentile
wavelet-HLL_firstorder_90Percentile
wavelet-HLL_firstorder_Energy
wavelet-HLL_firstorder_Entropy
wavelet-HLL_firstorder_InterquartileRange
wavelet-HLL_firstorder_Kurtosis
wavelet-HLL_firstorder_Maximum
wavelet-HLL_firstorder_MeanAbsoluteDeviation
wavelet-HLL_firstorder_Mean
wavelet-HLL_firstorder_Median
wavelet-HLL_firstorder_Minimum
wavelet-HLL_firstorder_Range
wavelet-HLL_firstorder_RobustMeanAbsoluteDeviation
wavelet-HLL_firstorder_RootMeanSquared
wavelet-HLL_firstorder_Skewness
wavelet-HLL_firstorder_TotalEnergy

wavelet-HLL_firstorder_Uniformity
wavelet-HLL_firstorder_Variance
wavelet-HLL_glcm_Autocorrelation
wavelet-HLL_glcm_ClusterProminence
wavelet-HLL_glcm_ClusterShade
wavelet-HLL_glcm_ClusterTendency
wavelet-HLL_glcm_Contrast
wavelet-HLL_glcm_Correlation
wavelet-HLL_glcm_DifferenceAverage
wavelet-HLL_glcm_DifferenceEntropy
wavelet-HLL_glcm_DifferenceVariance
wavelet-HLL_glcm_Id
wavelet-HLL_glcm_Idm
wavelet-HLL_glcm_Idmn
wavelet-HLL_glcm_Idn
wavelet-HLL_glcm_Imc1
wavelet-HLL_glcm_Imc2
wavelet-HLL_glcm_InverseVariance
wavelet-HLL_glcm_JointAverage
wavelet-HLL_glcm_JointEnergy
wavelet-HLL_glcm_JointEntropy
wavelet-HLL_glcm_MCC
wavelet-HLL_glcm_MaximumProbability
wavelet-HLL_glcm_SumAverage
wavelet-HLL_glcm_SumEntropy
wavelet-HLL_glcm_SumSquares
wavelet-HLL_gldm_DependenceEntropy
wavelet-HLL_gldm_DependenceNonUniformity
wavelet-HLL_gldm_DependenceNonUniformityNormalized
wavelet-HLL_gldm_DependenceVariance
wavelet-HLL_gldm_GrayLevelNonUniformity
wavelet-HLL_gldm_GrayLevelVariance
wavelet-HLL_gldm_HighGrayLevelEmphasis
wavelet-HLL_gldm_LargeDependenceEmphasis
wavelet-HLL_gldm_LargeDependenceHighGrayLevelEmphasis
wavelet-HLL_gldm_LargeDependenceLowGrayLevelEmphasis
wavelet-HLL_gldm_LowGrayLevelEmphasis
wavelet-HLL_gldm_SmallDependenceEmphasis
wavelet-HLL_gldm_SmallDependenceHighGrayLevelEmphasis
wavelet-HLL_gldm_SmallDependenceLowGrayLevelEmphasis
wavelet-HLL_glrlm_GrayLevelNonUniformity
wavelet-HLL_glrlm_GrayLevelNonUniformityNormalized
wavelet-HLL_glrlm_GrayLevelVariance
wavelet-HLL_glrlm_HighGrayLevelRunEmphasis
wavelet-HLL_glrlm_LongRunEmphasis
wavelet-HLL_glrlm_LongRunHighGrayLevelEmphasis
wavelet-HLL_glrlm_LongRunLowGrayLevelEmphasis
wavelet-HLL_glrlm_LowGrayLevelRunEmphasis
wavelet-HLL_glrlm_RunEntropy
wavelet-HLL_glrlm_RunLengthNonUniformity
wavelet-HLL_glrlm_RunLengthNonUniformityNormalized
wavelet-HLL_glrlm_RunPercentage
wavelet-HLL_glrlm_RunVariance

wavelet-HLL_glrlm_ShortRunEmphasis
wavelet-HLL_glrlm_ShortRunHighGrayLevelEmphasis
wavelet-HLL_glrlm_ShortRunLowGrayLevelEmphasis
wavelet-HLL_glszm_GrayLevelNonUniformity
wavelet-HLL_glszm_GrayLevelNonUniformityNormalized
wavelet-HLL_glszm_GrayLevelVariance
wavelet-HLL_glszm_HighGrayLevelZoneEmphasis
wavelet-HLL_glszm_LargeAreaEmphasis
wavelet-HLL_glszm_LargeAreaHighGrayLevelEmphasis
wavelet-HLL_glszm_LargeAreaLowGrayLevelEmphasis
wavelet-HLL_glszm_LowGrayLevelZoneEmphasis
wavelet-HLL_glszm_SizeZoneNonUniformity
wavelet-HLL_glszm_SizeZoneNonUniformityNormalized
wavelet-HLL_glszm_SmallAreaEmphasis
wavelet-HLL_glszm_SmallAreaHighGrayLevelEmphasis
wavelet-HLL_glszm_SmallAreaLowGrayLevelEmphasis
wavelet-HLL_glszm_ZoneEntropy
wavelet-HLL_glszm_ZonePercentage
wavelet-HLL_glszm_ZoneVariance
wavelet-HLL_ngtdm_Busyness
wavelet-HLL_ngtdm_Coarseness
wavelet-HLL_ngtdm_Complexity
wavelet-HLL_ngtdm_Contrast
wavelet-HLL_ngtdm_Strength
wavelet-HLH_firstorder_10Percentile
wavelet-HLH_firstorder_90Percentile
wavelet-HLH_firstorder_Energy
wavelet-HLH_firstorder_Entropy
wavelet-HLH_firstorder_InterquartileRange
wavelet-HLH_firstorder_Kurtosis
wavelet-HLH_firstorder_Maximum
wavelet-HLH_firstorder_MeanAbsoluteDeviation
wavelet-HLH_firstorder_Mean
wavelet-HLH_firstorder_Median
wavelet-HLH_firstorder_Minimum
wavelet-HLH_firstorder_Range
wavelet-HLH_firstorder_RobustMeanAbsoluteDeviation
wavelet-HLH_firstorder_RootMeanSquared
wavelet-HLH_firstorder_Skewness
wavelet-HLH_firstorder_TotalEnergy
wavelet-HLH_firstorder_Uniformity
wavelet-HLH_firstorder_Variance
wavelet-HLH_glcm_Autocorrelation
wavelet-HLH_glcm_ClusterProminence
wavelet-HLH_glcm_ClusterShade
wavelet-HLH_glcm_ClusterTendency
wavelet-HLH_glcm_Contrast
wavelet-HLH_glcm_Correlation
wavelet-HLH_glcm_DifferenceAverage
wavelet-HLH_glcm_DifferenceEntropy
wavelet-HLH_glcm_DifferenceVariance
wavelet-HLH_glcm_Id
wavelet-HLH_glcm_Idm

wavelet-HLH_glcm_Idmn
wavelet-HLH_glcm_Idn
wavelet-HLH_glcm_Imc1
wavelet-HLH_glcm_Imc2
wavelet-HLH_glcm_InverseVariance
wavelet-HLH_glcm_JointAverage
wavelet-HLH_glcm_JointEnergy
wavelet-HLH_glcm_JointEntropy
wavelet-HLH_glcm_MCC
wavelet-HLH_glcm_MaximumProbability
wavelet-HLH_glcm_SumAverage
wavelet-HLH_glcm_SumEntropy
wavelet-HLH_glcm_SumSquares
wavelet-HLH_gldm_DependenceEntropy
wavelet-HLH_gldm_DependenceNonUniformity
wavelet-HLH_gldm_DependenceNonUniformityNormalized
wavelet-HLH_gldm_DependenceVariance
wavelet-HLH_gldm_GrayLevelNonUniformity
wavelet-HLH_gldm_GrayLevelVariance
wavelet-HLH_gldm_HighGrayLevelEmphasis
wavelet-HLH_gldm_LargeDependenceEmphasis
wavelet-HLH_gldm_LargeDependenceHighGrayLevelEmphasis
wavelet-HLH_gldm_LargeDependenceLowGrayLevelEmphasis
wavelet-HLH_gldm_LowGrayLevelEmphasis
wavelet-HLH_gldm_SmallDependenceEmphasis
wavelet-HLH_gldm_SmallDependenceHighGrayLevelEmphasis
wavelet-HLH_gldm_SmallDependenceLowGrayLevelEmphasis
wavelet-HLH_glrlm_GrayLevelNonUniformity
wavelet-HLH_glrlm_GrayLevelNonUniformityNormalized
wavelet-HLH_glrlm_GrayLevelVariance
wavelet-HLH_glrlm_HighGrayLevelRunEmphasis
wavelet-HLH_glrlm_LongRunEmphasis
wavelet-HLH_glrlm_LongRunHighGrayLevelEmphasis
wavelet-HLH_glrlm_LongRunLowGrayLevelEmphasis
wavelet-HLH_glrlm_LowGrayLevelRunEmphasis
wavelet-HLH_glrlm_RunEntropy
wavelet-HLH_glrlm_RunLengthNonUniformity
wavelet-HLH_glrlm_RunLengthNonUniformityNormalized
wavelet-HLH_glrlm_RunPercentage
wavelet-HLH_glrlm_RunVariance
wavelet-HLH_glrlm_ShortRunEmphasis
wavelet-HLH_glrlm_ShortRunHighGrayLevelEmphasis
wavelet-HLH_glrlm_ShortRunLowGrayLevelEmphasis
wavelet-HLH_glszm_GrayLevelNonUniformity
wavelet-HLH_glszm_GrayLevelNonUniformityNormalized
wavelet-HLH_glszm_GrayLevelVariance
wavelet-HLH_glszm_HighGrayLevelZoneEmphasis
wavelet-HLH_glszm_LargeAreaEmphasis
wavelet-HLH_glszm_LargeAreaHighGrayLevelEmphasis
wavelet-HLH_glszm_LargeAreaLowGrayLevelEmphasis
wavelet-HLH_glszm_LowGrayLevelZoneEmphasis
wavelet-HLH_glszm_SizeZoneNonUniformity
wavelet-HLH_glszm_SizeZoneNonUniformityNormalized

wavelet-HLH_glszm_SmallAreaEmphasis
wavelet-HLH_glszm_SmallAreaHighGrayLevelEmphasis
wavelet-HLH_glszm_SmallAreaLowGrayLevelEmphasis
wavelet-HLH_glszm_ZoneEntropy
wavelet-HLH_glszm_ZonePercentage
wavelet-HLH_glszm_ZoneVariance
wavelet-HLH_ngtdm_Busyness
wavelet-HLH_ngtdm_Coarseness
wavelet-HLH_ngtdm_Complexity
wavelet-HLH_ngtdm_Contrast
wavelet-HLH_ngtdm_Strength
wavelet-HHL_firstorder_10Percentile
wavelet-HHL_firstorder_90Percentile
wavelet-HHL_firstorder_Energy
wavelet-HHL_firstorder_Entropy
wavelet-HHL_firstorder_InterquartileRange
wavelet-HHL_firstorder_Kurtosis
wavelet-HHL_firstorder_Maximum
wavelet-HHL_firstorder_MeanAbsoluteDeviation
wavelet-HHL_firstorder_Mean
wavelet-HHL_firstorder_Median
wavelet-HHL_firstorder_Minimum
wavelet-HHL_firstorder_Range
wavelet-HHL_firstorder_RobustMeanAbsoluteDeviation
wavelet-HHL_firstorder_RootMeanSquared
wavelet-HHL_firstorder_Skewness
wavelet-HHL_firstorder_TotalEnergy
wavelet-HHL_firstorder_Uniformity
wavelet-HHL_firstorder_Variance
wavelet-HHL_glcm_Autocorrelation
wavelet-HHL_glcm_ClusterProminence
wavelet-HHL_glcm_ClusterShade
wavelet-HHL_glcm_ClusterTendency
wavelet-HHL_glcm_Contrast
wavelet-HHL_glcm_Correlation
wavelet-HHL_glcm_DifferenceAverage
wavelet-HHL_glcm_DifferenceEntropy
wavelet-HHL_glcm_DifferenceVariance
wavelet-HHL_glcm_Id
wavelet-HHL_glcm_Idm
wavelet-HHL_glcm_Idmn
wavelet-HHL_glcm_Idn
wavelet-HHL_glcm_Imc1
wavelet-HHL_glcm_Imc2
wavelet-HHL_glcm_InverseVariance
wavelet-HHL_glcm_JointAverage
wavelet-HHL_glcm_JointEnergy
wavelet-HHL_glcm_JointEntropy
wavelet-HHL_glcm_MCC
wavelet-HHL_glcm_MaximumProbability
wavelet-HHL_glcm_SumAverage
wavelet-HHL_glcm_SumEntropy
wavelet-HHL_glcm_SumSquares

wavelet-HHL_gldm_DependenceEntropy
wavelet-HHL_gldm_DependenceNonUniformity
wavelet-HHL_gldm_DependenceNonUniformityNormalized
wavelet-HHL_gldm_DependenceVariance
wavelet-HHL_gldm_GrayLevelNonUniformity
wavelet-HHL_gldm_GrayLevelVariance
wavelet-HHL_gldm_HighGrayLevelEmphasis
wavelet-HHL_gldm_LargeDependenceEmphasis
wavelet-HHL_gldm_LargeDependenceHighGrayLevelEmphasis
wavelet-HHL_gldm_LargeDependenceLowGrayLevelEmphasis
wavelet-HHL_gldm_LowGrayLevelEmphasis
wavelet-HHL_gldm_SmallDependenceEmphasis
wavelet-HHL_gldm_SmallDependenceHighGrayLevelEmphasis
wavelet-HHL_gldm_SmallDependenceLowGrayLevelEmphasis
wavelet-HHL_glrlm_GrayLevelNonUniformity
wavelet-HHL_glrlm_GrayLevelNonUniformityNormalized
wavelet-HHL_glrlm_GrayLevelVariance
wavelet-HHL_glrlm_HighGrayLevelRunEmphasis
wavelet-HHL_glrlm_LongRunEmphasis
wavelet-HHL_glrlm_LongRunHighGrayLevelEmphasis
wavelet-HHL_glrlm_LongRunLowGrayLevelEmphasis
wavelet-HHL_glrlm_LowGrayLevelRunEmphasis
wavelet-HHL_glrlm_RunEntropy
wavelet-HHL_glrlm_RunLengthNonUniformity
wavelet-HHL_glrlm_RunLengthNonUniformityNormalized
wavelet-HHL_glrlm_RunPercentage
wavelet-HHL_glrlm_RunVariance
wavelet-HHL_glrlm_ShortRunEmphasis
wavelet-HHL_glrlm_ShortRunHighGrayLevelEmphasis
wavelet-HHL_glrlm_ShortRunLowGrayLevelEmphasis
wavelet-HHL_glszm_GrayLevelNonUniformity
wavelet-HHL_glszm_GrayLevelNonUniformityNormalized
wavelet-HHL_glszm_GrayLevelVariance
wavelet-HHL_glszm_HighGrayLevelZoneEmphasis
wavelet-HHL_glszm_LargeAreaEmphasis
wavelet-HHL_glszm_LargeAreaHighGrayLevelEmphasis
wavelet-HHL_glszm_LargeAreaLowGrayLevelEmphasis
wavelet-HHL_glszm_LowGrayLevelZoneEmphasis
wavelet-HHL_glszm_SizeZoneNonUniformity
wavelet-HHL_glszm_SizeZoneNonUniformityNormalized
wavelet-HHL_glszm_SmallAreaEmphasis
wavelet-HHL_glszm_SmallAreaHighGrayLevelEmphasis
wavelet-HHL_glszm_SmallAreaLowGrayLevelEmphasis
wavelet-HHL_glszm_ZoneEntropy
wavelet-HHL_glszm_ZonePercentage
wavelet-HHL_glszm_ZoneVariance
wavelet-HHL_ngtdm_Busyness
wavelet-HHL_ngtdm_Coarseness
wavelet-HHL_ngtdm_Complexity
wavelet-HHL_ngtdm_Contrast
wavelet-HHL_ngtdm_Strength
wavelet-HHH_firstorder_10Percentile
wavelet-HHH_firstorder_90Percentile

wavelet-HHH_firstorder_Energy
wavelet-HHH_firstorder_Entropy
wavelet-HHH_firstorder_InterquartileRange
wavelet-HHH_firstorder_Kurtosis
wavelet-HHH_firstorder_Maximum
wavelet-HHH_firstorder_MeanAbsoluteDeviation
wavelet-HHH_firstorder_Mean
wavelet-HHH_firstorder_Median
wavelet-HHH_firstorder_Minimum
wavelet-HHH_firstorder_Range
wavelet-HHH_firstorder_RobustMeanAbsoluteDeviation
wavelet-HHH_firstorder_RootMeanSquared
wavelet-HHH_firstorder_Skewness
wavelet-HHH_firstorder_TotalEnergy
wavelet-HHH_firstorder_Uniformity
wavelet-HHH_firstorder_Variance
wavelet-HHH_glcm_Autocorrelation
wavelet-HHH_glcm_ClusterProminence
wavelet-HHH_glcm_ClusterShade
wavelet-HHH_glcm_ClusterTendency
wavelet-HHH_glcm_Contrast
wavelet-HHH_glcm_Correlation
wavelet-HHH_glcm_DifferenceAverage
wavelet-HHH_glcm_DifferenceEntropy
wavelet-HHH_glcm_DifferenceVariance
wavelet-HHH_glcm_Id
wavelet-HHH_glcm_Idm
wavelet-HHH_glcm_Idmn
wavelet-HHH_glcm_Idn
wavelet-HHH_glcm_Imc1
wavelet-HHH_glcm_Imc2
wavelet-HHH_glcm_InverseVariance
wavelet-HHH_glcm_JointAverage
wavelet-HHH_glcm_JointEnergy
wavelet-HHH_glcm_JointEntropy
wavelet-HHH_glcm_MCC
wavelet-HHH_glcm_MaximumProbability
wavelet-HHH_glcm_SumAverage
wavelet-HHH_glcm_SumEntropy
wavelet-HHH_glcm_SumSquares
wavelet-HHH_gldm_DependenceEntropy
wavelet-HHH_gldm_DependenceNonUniformity
wavelet-HHH_gldm_DependenceNonUniformityNormalized
wavelet-HHH_gldm_DependenceVariance
wavelet-HHH_gldm_GrayLevelNonUniformity
wavelet-HHH_gldm_GrayLevelVariance
wavelet-HHH_gldm_HighGrayLevelEmphasis
wavelet-HHH_gldm_LargeDependenceEmphasis
wavelet-HHH_gldm_LargeDependenceHighGrayLevelEmphasis
wavelet-HHH_gldm_LargeDependenceLowGrayLevelEmphasis
wavelet-HHH_gldm_LowGrayLevelEmphasis
wavelet-HHH_gldm_SmallDependenceEmphasis
wavelet-HHH_gldm_SmallDependenceHighGrayLevelEmphasis

wavelet-HHH_gldm_SmallDependenceLowGrayLevelEmphasis
wavelet-HHH_glrlm_GrayLevelNonUniformity
wavelet-HHH_glrlm_GrayLevelNonUniformityNormalized
wavelet-HHH_glrlm_GrayLevelVariance
wavelet-HHH_glrlm_HighGrayLevelRunEmphasis
wavelet-HHH_glrlm_LongRunEmphasis
wavelet-HHH_glrlm_LongRunHighGrayLevelEmphasis
wavelet-HHH_glrlm_LongRunLowGrayLevelEmphasis
wavelet-HHH_glrlm_LowGrayLevelRunEmphasis
wavelet-HHH_glrlm_RunEntropy
wavelet-HHH_glrlm_RunLengthNonUniformity
wavelet-HHH_glrlm_RunLengthNonUniformityNormalized
wavelet-HHH_glrlm_RunPercentage
wavelet-HHH_glrlm_RunVariance
wavelet-HHH_glrlm_ShortRunEmphasis
wavelet-HHH_glrlm_ShortRunHighGrayLevelEmphasis
wavelet-HHH_glrlm_ShortRunLowGrayLevelEmphasis
wavelet-HHH_glszm_GrayLevelNonUniformity
wavelet-HHH_glszm_GrayLevelNonUniformityNormalized
wavelet-HHH_glszm_GrayLevelVariance
wavelet-HHH_glszm_HighGrayLevelZoneEmphasis
wavelet-HHH_glszm_LargeAreaEmphasis
wavelet-HHH_glszm_LargeAreaHighGrayLevelEmphasis
wavelet-HHH_glszm_LargeAreaLowGrayLevelEmphasis
wavelet-HHH_glszm_LowGrayLevelZoneEmphasis
wavelet-HHH_glszm_SizeZoneNonUniformity
wavelet-HHH_glszm_SizeZoneNonUniformityNormalized
wavelet-HHH_glszm_SmallAreaEmphasis
wavelet-HHH_glszm_SmallAreaHighGrayLevelEmphasis
wavelet-HHH_glszm_SmallAreaLowGrayLevelEmphasis
wavelet-HHH_glszm_ZoneEntropy
wavelet-HHH_glszm_ZonePercentage
wavelet-HHH_glszm_ZoneVariance
wavelet-HHH_ngtdm_Busyness
wavelet-HHH_ngtdm_Coarseness
wavelet-HHH_ngtdm_Complexity
wavelet-HHH_ngtdm_Contrast
wavelet-HHH_ngtdm_Strength
wavelet-LLL_firstorder_10Percentile
wavelet-LLL_firstorder_90Percentile
wavelet-LLL_firstorder_Energy
wavelet-LLL_firstorder_Entropy
wavelet-LLL_firstorder_InterquartileRange
wavelet-LLL_firstorder_Kurtosis
wavelet-LLL_firstorder_Maximum
wavelet-LLL_firstorder_MeanAbsoluteDeviation
wavelet-LLL_firstorder_Mean
wavelet-LLL_firstorder_Median
wavelet-LLL_firstorder_Minimum
wavelet-LLL_firstorder_Range
wavelet-LLL_firstorder_RobustMeanAbsoluteDeviation
wavelet-LLL_firstorder_RootMeanSquared
wavelet-LLL_firstorder_Skewness

wavelet-LLL_firstorder_TotalEnergy
wavelet-LLL_firstorder_Uniformity
wavelet-LLL_firstorder_Variance
wavelet-LLL_glcm_Autocorrelation
wavelet-LLL_glcm_ClusterProminence
wavelet-LLL_glcm_ClusterShade
wavelet-LLL_glcm_ClusterTendency
wavelet-LLL_glcm_Contrast
wavelet-LLL_glcm_Correlation
wavelet-LLL_glcm_DifferenceAverage
wavelet-LLL_glcm_DifferenceEntropy
wavelet-LLL_glcm_DifferenceVariance
wavelet-LLL_glcm_Id
wavelet-LLL_glcm_Idm
wavelet-LLL_glcm_Idmn
wavelet-LLL_glcm_Idn
wavelet-LLL_glcm_Imc1
wavelet-LLL_glcm_Imc2
wavelet-LLL_glcm_InverseVariance
wavelet-LLL_glcm_JointAverage
wavelet-LLL_glcm_JointEnergy
wavelet-LLL_glcm_JointEntropy
wavelet-LLL_glcm_MCC
wavelet-LLL_glcm_MaximumProbability
wavelet-LLL_glcm_SumAverage
wavelet-LLL_glcm_SumEntropy
wavelet-LLL_glcm_SumSquares
wavelet-LLL_gldm_DependenceEntropy
wavelet-LLL_gldm_DependenceNonUniformity
wavelet-LLL_gldm_DependenceNonUniformityNormalized
wavelet-LLL_gldm_DependenceVariance
wavelet-LLL_gldm_GrayLevelNonUniformity
wavelet-LLL_gldm_GrayLevelVariance
wavelet-LLL_gldm_HighGrayLevelEmphasis
wavelet-LLL_gldm_LargeDependenceEmphasis
wavelet-LLL_gldm_LargeDependenceHighGrayLevelEmphasis
wavelet-LLL_gldm_LargeDependenceLowGrayLevelEmphasis
wavelet-LLL_gldm_LowGrayLevelEmphasis
wavelet-LLL_gldm_SmallDependenceEmphasis
wavelet-LLL_gldm_SmallDependenceHighGrayLevelEmphasis
wavelet-LLL_gldm_SmallDependenceLowGrayLevelEmphasis
wavelet-LLL_glrlm_GrayLevelNonUniformity
wavelet-LLL_glrlm_GrayLevelNonUniformityNormalized
wavelet-LLL_glrlm_GrayLevelVariance
wavelet-LLL_glrlm_HighGrayLevelRunEmphasis
wavelet-LLL_glrlm_LongRunEmphasis
wavelet-LLL_glrlm_LongRunHighGrayLevelEmphasis
wavelet-LLL_glrlm_LongRunLowGrayLevelEmphasis
wavelet-LLL_glrlm_LowGrayLevelRunEmphasis
wavelet-LLL_glrlm_RunEntropy
wavelet-LLL_glrlm_RunLengthNonUniformity
wavelet-LLL_glrlm_RunLengthNonUniformityNormalized
wavelet-LLL_glrlm_RunPercentage

wavelet-LLL_glrlm_RunVariance
wavelet-LLL_glrlm_ShortRunEmphasis
wavelet-LLL_glrlm_ShortRunHighGrayLevelEmphasis
wavelet-LLL_glrlm_ShortRunLowGrayLevelEmphasis
wavelet-LLL_glszm_GrayLevelNonUniformity
wavelet-LLL_glszm_GrayLevelNonUniformityNormalized
wavelet-LLL_glszm_GrayLevelVariance
wavelet-LLL_glszm_HighGrayLevelZoneEmphasis
wavelet-LLL_glszm_LargeAreaEmphasis
wavelet-LLL_glszm_LargeAreaHighGrayLevelEmphasis
wavelet-LLL_glszm_LargeAreaLowGrayLevelEmphasis
wavelet-LLL_glszm_LowGrayLevelZoneEmphasis
wavelet-LLL_glszm_SizeZoneNonUniformity
wavelet-LLL_glszm_SizeZoneNonUniformityNormalized
wavelet-LLL_glszm_SmallAreaEmphasis
wavelet-LLL_glszm_SmallAreaHighGrayLevelEmphasis
wavelet-LLL_glszm_SmallAreaLowGrayLevelEmphasis
wavelet-LLL_glszm_ZoneEntropy
wavelet-LLL_glszm_ZonePercentage
wavelet-LLL_glszm_ZoneVariance
wavelet-LLL_ngtdm_Busyness
wavelet-LLL_ngtdm_Coarseness
wavelet-LLL_ngtdm_Complexity
wavelet-LLL_ngtdm_Contrast
wavelet-LLL_ngtdm_Strength

Figure S1 - AUC of radiomic and clinical-radiomic models (without cross-validation) for prediction of post-treatment abnormal DLco

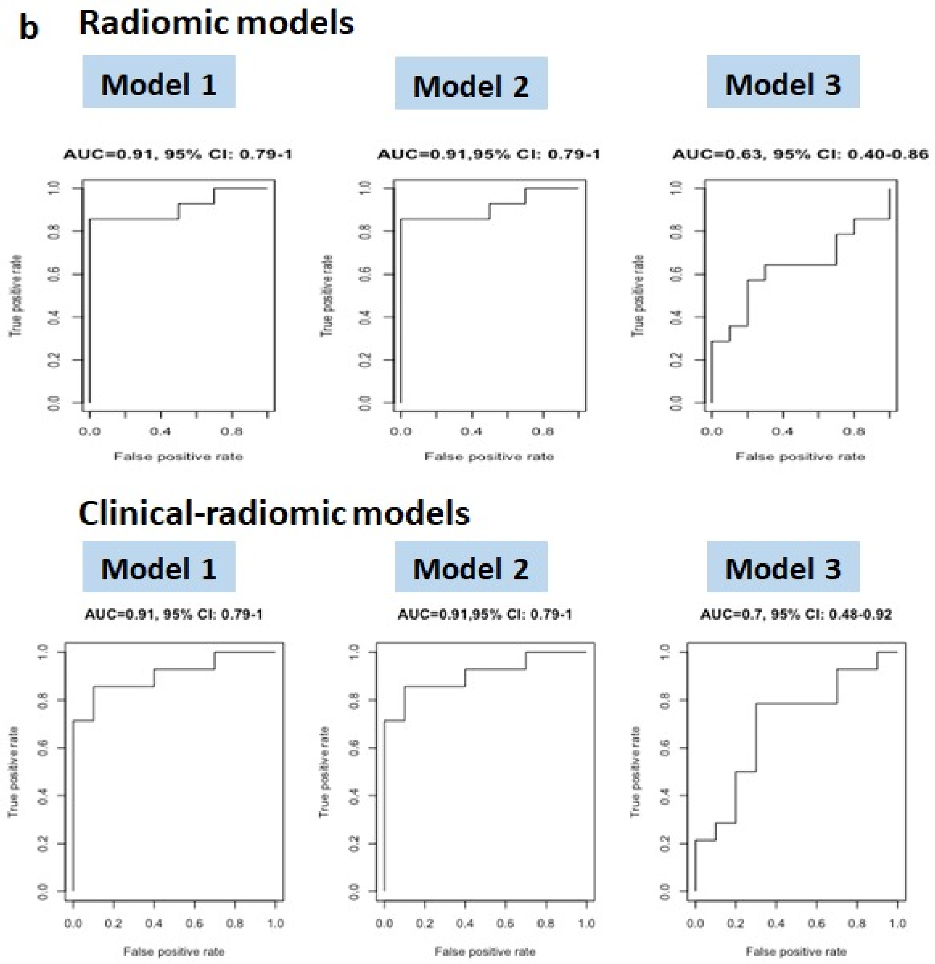

Supplementary material Table S3 -: Median value of repeated cross validation AUC for models predicting abnormal baseline and post-treatment DL$_{CO}$

		Repeated cross validation of AUC (median, IQR)
Radiomic Models	Model 1	0.94 (0.90 - 1.0)
	Model 2	0.94 (0.90 - 1.0)
	Model 3	0.67 (0.60 - 0.70)
Clinical-Radiomic Models	Model 1	0.88 (0.83 - 0.92)
	Model 2	0.88 (0.83 - 0.92)
	Model 3	0.67 (0.60 - 0.71)
Clinical Model		\
